# Disruption of DNA methylation underpins the neuroinflammation induced by targeted CNS radiotherapy

**DOI:** 10.1093/brain/awaf163

**Published:** 2025-04-29

**Authors:** Thomas O Millner, Pratistha Panday, Yunchen Xiao, James G Nicholson, James R Boot, Zsharmaine Arpe, Paul A Stevens, Nadia N Rahman, Xinyu Zhang, Charles Mein, Neil Kitchen, Andrew W McEvoy, Edward McKintosh, Grainne S McKenna, Dimitrios Paraskevopoulos, Nicolae Radu Zabet, Rachel Lewis, Sara Badodi, Silvia Marino

**Affiliations:** Blizard Institute, Queen Mary University of London, London E1 2AT, UK; Barts Brain Tumour Centre, Barts Health NHS Trust, London E1 1BB, UK; National Hospital for Neurology and Neurosurgery, UCLH NHS Trust, London WC1N 3BG, UK; Barts Cancer Institute, Queen Mary University of London, London EC1M 6AU, UK; The Royal London Hospital, Barts Health NHS Trust, London E1 1FR, UK; Blizard Institute, Queen Mary University of London, London E1 2AT, UK; Barts Brain Tumour Centre, Barts Health NHS Trust, London E1 1BB, UK; Blizard Institute, Queen Mary University of London, London E1 2AT, UK; Blizard Institute, Queen Mary University of London, London E1 2AT, UK; Blizard Institute, Queen Mary University of London, London E1 2AT, UK; National Hospital for Neurology and Neurosurgery, UCLH NHS Trust, London WC1N 3BG, UK; Blizard Institute, Queen Mary University of London, London E1 2AT, UK; Barts Cancer Institute, Queen Mary University of London, London EC1M 6AU, UK; Blizard Institute, Queen Mary University of London, London E1 2AT, UK; Blizard Institute, Queen Mary University of London, London E1 2AT, UK; National Hospital for Neurology and Neurosurgery, UCLH NHS Trust, London WC1N 3BG, UK; National Hospital for Neurology and Neurosurgery, UCLH NHS Trust, London WC1N 3BG, UK; Barts Brain Tumour Centre, Barts Health NHS Trust, London E1 1BB, UK; The Royal London Hospital, Barts Health NHS Trust, London E1 1FR, UK; Barts Brain Tumour Centre, Barts Health NHS Trust, London E1 1BB, UK; The Royal London Hospital, Barts Health NHS Trust, London E1 1FR, UK; Blizard Institute, Queen Mary University of London, London E1 2AT, UK; Barts Brain Tumour Centre, Barts Health NHS Trust, London E1 1BB, UK; The Royal London Hospital, Barts Health NHS Trust, London E1 1FR, UK; Blizard Institute, Queen Mary University of London, London E1 2AT, UK; Barts Brain Tumour Centre, Barts Health NHS Trust, London E1 1BB, UK; The Royal London Hospital, Barts Health NHS Trust, London E1 1FR, UK; Blizard Institute, Queen Mary University of London, London E1 2AT, UK; Blizard Institute, Queen Mary University of London, London E1 2AT, UK; Barts Brain Tumour Centre, Barts Health NHS Trust, London E1 1BB, UK; National Hospital for Neurology and Neurosurgery, UCLH NHS Trust, London WC1N 3BG, UK; Barts Cancer Institute, Queen Mary University of London, London EC1M 6AU, UK; The Royal London Hospital, Barts Health NHS Trust, London E1 1FR, UK

**Keywords:** radiotherapy, epigenetics, neuroinflammation, neuro-oncology, DNA methylation, spatial transcriptomics

## Abstract

Targeted radiotherapy is integral to the increasing survival of cancer patients; however, it has significant side effects, the underlying cellular and molecular mechanisms of which are ill-defined. It is well documented that targeted radiotherapy induces epigenetic changes in neoplastic tissue, which impacts tumour evolution; however, whether epigenetic deregulation also occurs in the surrounding non-neoplastic tissue and contributes to the occurrence of side effects is unknown.

We characterized the DNA methylome in a unique cohort of irradiated peri-lesional brain tissue samples and integrated it with gene expression analysis at the spatial level. We show differences in DNA methylation patterns in irradiated brain tissue and identify specific inflammatory micro-environmental niches and their regulatory neuropeptides after irradiation. Finally, we show in a cerebral organoid model, that the same neuropeptides are upregulated as well as similar DNA methylation alterations and disruption of the DNA methylation machinery, in keeping with the interpretation that epigenetic dysregulation plays a role in neurotoxicity, hence raising the possibility it could represent a novel target for the reduction of radiotherapy side effects.

## Teaser

By examining brain tissue from patients who have received radiotherapy and comparing the findings with a cerebral organoid model, Millner *et al.* obtain evidence that post-radiotherapy neuroinflammation is linked to changes in DNA methylation. Targeting these changes could be a promising approach to reduce radiotherapy side effects.

**See Kular et al. (https://doi.org/10.1093/brain/awaf270) for a scientific commentary on this article.**

## Introduction

There are ∼20 million new cases of cancer per year worldwide, with ∼10 million deaths, and rates are rising.^1^ Advances in primary site control have increased survival; therefore, the management of metastases is becoming increasingly important. The estimated incidence of brain metastasis is ∼15%, although this is likely conservative.^2^ Targeted radiotherapy (RT) is essential to the treatment of brain metastases, as well as vascular malformations and other focal neurological diseases, but can have significant side effects. Short-term side effects (0–6 months) of RT include tiredness, nausea, headaches and local hair loss, whilst long-term side effects (>6 months) include cognitive impairment, severe headaches, radiation necrosis and even the development of secondary tumours.^3^ The mechanisms underlying RT-induced neurotoxicity have been studied in animal models and are suggested to include senescence and apoptosis of neurons, endothelial cells and glia, neuroinflammation and disruption of the blood–brain barrier, with their molecular underpinnings and translational value in humans yet to be fully assessed.^4,5^ This is an essential first step to enable the identification of agents that may offer protection to normal brain tissue and preserve its functional integrity while not hampering the effects on tumour cells.

Radiation kills tumour cells by inducing DNA damage, by the direct action of radiation and by the indirect action of reactive oxygen species generation on DNA. Radiation is also a potent epigenotoxic stressor^6^ and has been associated with changes in DNA methylation (DNAme), histone dynamics and modulation of non-coding RNAs.^7^ DNAme alterations are the best characterized of these changes, but reported results are highly variable, depending on the type of radiation, radiation dose and biological context.^8,9^ The epigenetic modifications that occur in irradiated neoplastic tissue have been explored^10,11^; however, there are no studies to our knowledge that have assessed the changes in DNAme induced by irradiation in human brain.

Here, we harnessed human samples from patients who have undergone targeted radiotherapy, and utilized bulk DNAme and RNA-sequencing (RNAseq) in combination with spatial transcriptomics, as well as a cerebral organoid (CO) model system, to characterize the impact on DNAme and its phenotypic transcriptional consequences in the human brain after targeted RT.

## Materials and methods

The ‘Materials and methods’ are detailed in the Supplementary material.

## Results

### DNA methylation changes impact gene expression in irradiated peri-lesional brain tissue

To explore the molecular mechanisms underlying RT-induced neurotoxicity, we retrospectively identified neurosurgical samples from patients who had undergone targeted RT to a brain lesion, followed by resection of the same area. We included cases that contained peri-lesional brain tissue within the RT treatment field. Of the 14 cases identified, 9 were treated for brain metastases and 5 for other conditions (Table 1). Twelve samples of peri-lesional brain tissue surrounding metastases from RT-naïve patients were analysed as controls. The time of resection post-irradiation was 7–240 months, reflecting a range of clinically relevant time points. Histology showed well-described^12^ changes: reactive gliosis; chronic inflammation; vascular changes in the white matter and cortex; and rare areas of white matter rarefaction and necrosis (Supplementary Fig. 1A–D).

**Table 1:** Patient sample clinical characteristics

Sample	Sex	Age	Lesion	Location of lesion	Type of RT (dose/No.)	Time RT-resection (months)	ST analysis
RT1	Female	24	AVM	Parietal	GK	65	X
RT2	Male	57	H and N cancer (SCC)	Frontal	IMRT (65 Gy/30)	43	X
RT3	Female	44	AVM	Frontal	GK	240	X
RT4	Male	61	Metastasis (L)	Frontal	GK	9	–
RT5	Female	62	AVM	Frontal	GK	28	X
RT6	Female	60	Metastasis (L)	Frontal	CK	13	X
RT7	Female	38	Metastasis (M)	Parietal	GK	18	–
RT8	Male	64	Metastasis (L)	Frontal	CK	9	X
RT9	Female	46	Metastasis (L)	Parietal	CK	28	X
RT10	Female	82	Metastasis (L)	Parietal	CK	24	X
RT11	Male	57	Metastasis (L)	Frontal	GK (18 Gy)	7	–
RT12	Female	59	Masson’s tumour/mesial temporal sclerosis	Temporal	GK	180	–
RT13	Male	65	H and N cancer (NK)	Frontal	NK	36	X
RT14	Female	45	AVM	Occipital	GK	180	X
Cnt1	Male	66	Metastasis (L)	Parietal	n/a	n/a	–
Cnt2	Female	84	Metastasis (L)	Frontal	n/a	n/a	X
Cnt3	Female	48	Metastasis (B)	Frontal	n/a	n/a	X
Cnt4	Female	73	Metastasis (NE)	Frontal	n/a	n/a	X
Cnt5	Female	73	Metastasis (L)	Frontal	n/a	n/a	–
Cnt6	Female	57	Metastasis (M)	Frontal	n/a	n/a	–
Cnt7	Male	53	Metastasis (L)	Temporal	n/a	n/a	–
Cnt8	Female	46	Metastasis (C)	Parietal	n/a	n/a	X
Cnt9	Male	64	Metastais (L)	Parietal	n/a	n/a	–
Cnt10	Female	55	Metastasis (L)	Frontal	n/a	n/a	–
Cnt11	Female	47	Metastasis (B)	Occipital	n/a	n/a	X
Cnt12	Male	54	Metastasis (L)	Frontal	n/a	n/a	–

Dose was a routine targeted radiotherapy dose (∼24 Gy) in single fraction unless specifically stated.

AVM = arteriovenous malformation; B = breast primary tumour; C = cervical primary tumour; CK = CyberKnife; GK = GammaKnife; H and N = head and neck; IMRT = intensity-modulated radiation therapy; L = lung primary tumour; M = melanoma primary tumour; n/a = not applicable; NE = neuroendocrine primary tumour; NK = not known; RT = radiotherapy; SCC = squamous cell carcinoma primary tumour.

**Figure 1:**
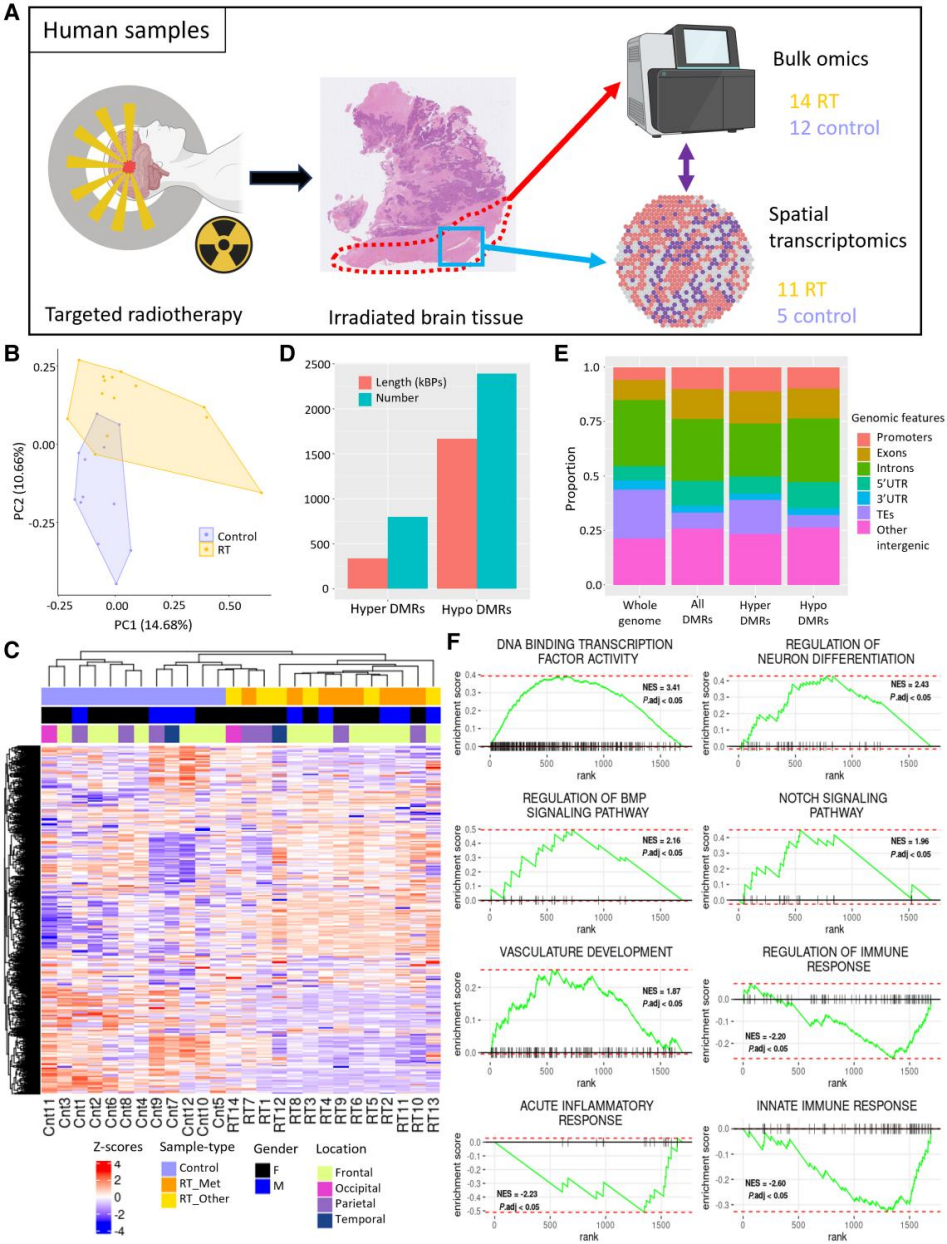
**Targeted radiotherapy drives differences in the DNA methylome and transcriptome of human peri-lesional brain tissue**. (**A**) Summary of the experimental pipeline for human tissue analysis; created in BioRender. Marino, S. (2025) https://BioRender.com/rgn40vq. (**B**) Principal component analysis of bulk DNA methylation data using all probes after standard filtering. (**C**) Heat map dendrogram for the 4000 most variable differentially methylated probes (DMPs) identified from the FFPE samples. Both row and column clustering were performed (see main text and Table 1 for details on RT_Met and RT_Other). (**D**) Bar plot showing number and length (in kilobase pairs) of hyper- and hypomethylated DMRs. (**E**) Bar plot showing genomic location of DMRs as a percentage. *Left* bar represents the whole genome as represented on the DNA methylation array. (**F**) Selected Gene Set Enrichment Analysis plots for all differentially methylated genes. DMR = differentially methylated region; FFPE = formalin-fixed paraffin-embedded; NES = normalized enrichment score.

Peri-lesional brain tissue was micro-dissected and DNAme profiling and RNA-seq performed (Fig. 1A). Referenced-based deconvolution of DNAme data showed irradiated samples had increased microglia/macrophages but fewer peripheral immune cells and fewer endothelial cells (Supplementary Fig. 1E and F), consistent with the histological features. Control samples had increased numbers of peripheral immune cells, reflective of the inflammation seen in the micro-environment of brain metastases even in the absence of RT.

Principal component analysis (PCA) of all DNAme array probes showed distinct clustering of irradiated and non-irradiated samples (Fig. 1B). Differentially methylated probes (DMPs) were then identified using potential confounding factors as covariates (see Supplementary material, ‘Materials and methods’ section). PCA findings were validated by hierarchical clustering with *z*-scores of the most variable probes (Fig. 1C). From the DMPs, 3194 differentially methylated regions (DMRs) were annotated. Overall, 75% (2392/3194) of the DMRs were hypomethylated in irradiated tissue (Fig. 1D), and this tendency towards hypomethylation was observed across all genomic locations except transposable elements (TEs; Supplementary Fig. 2A). Both hypomethylated and hypermethylated DMRs were enriched in promoters and exons; all DMRs together and hypomethylated DMRs alone were enriched in 5′ untranslated regions (UTRs) and intergenic regions, whilst hypomethylated and hypermethylated DMRs were depleted in TEs, and hypermethylated DMRs alone were depleted in introns (Fig. 1E and Supplementary material).

Next, we mapped DMRs to 2259 differentially methylated genes (DMGs). Pathway analysis of the top 150 promoter-hypomethylated DMGs highlighted pathways including DNA binding, neuronal fate specification and migration as well as FGF and BMP signalling pathways (Supplementary Fig. 2B), whilst the top 150 promoter-hypermethylated DMGs showed enrichment of pathways including complement activation and ion channel activity (Supplementary Fig. 2B). Gene Set Enrichment Analysis (GSEA) of all DMGs correspondingly highlighted pathways involved in DNA binding, neuronal regulation, BMP and NOTCH signalling, whilst acute immune responses showed negative enrichment (Fig. 1F). The specific pathways highlighted here all have reported functions in regulating neuroinflammation in various disease settings^13-15^ but have not previously been identified in the context of brain irradiation.

Analysis of the RNAseq data identified 1494 differentially expressed genes (DEGs; Supplementary Fig. 2C), of which 220 overlapped with DMGs (Supplementary Fig. 2D). Integration of DNAme and RNAseq datasets identified 115 concordant genes, defined as those genes that had anti-correlated expression and promoter/5′UTR/exon methylation and correlated expression and 3′UTR/intron/intergenic methylation (Supplementary Fig. 2E and F and see Supplementary material, ‘Materials and methods’ section for further details). Pathway analysis of these genes highlighted several developmental pathways related to CNS and mesenchymal cells, as well as pathways related to intermediate filaments and ion transport (Supplementary Fig. 2G). Within these concordant genes were those with known roles in neuroprotection and neuroinflammation, including *NPY*, *CLU* and *CRYAB*. Many transcription factors were also concordant (*HOXB3/6/7*, *HOXC4/6*, *IRX2/3/5*, *PAX5/6*, *TBX2/3*), suggesting a broad effect on cell phenotypes.

In summary, we have shown that targeted irradiation of brain leads to DNAme alterations across the genome, with resulting DMRs being predominantly hypomethylated and enriched at promoters, exons and 5′UTRs, suggesting an impact on gene expression. Examining the DMGs and genes with concordant methylation and expression, we found numerous transcription factors involved, likely to have broad phenotypic consequences, as well as pathways and genes with roles in neuroinflammation and response to brain injury.

### Spatial transcriptomics allows characterization of micro-environmental niches in irradiated brain

To investigate the transcriptome level changes post-RT in more detail, we derived spatial transcriptomic (ST) data with the Visium platform from 11 RT and 5 control cases (Table 1). Microscopy images enabled the identification of areas within the radiation field (<15 mm from lesion), which contained cortex and white matter where possible (two RT cases consisted only of white matter, one RT case only cortical tissue and one control case only cortical tissue). The anatomical location of samples was evenly represented across groups (Table 1).

**Figure 2:**
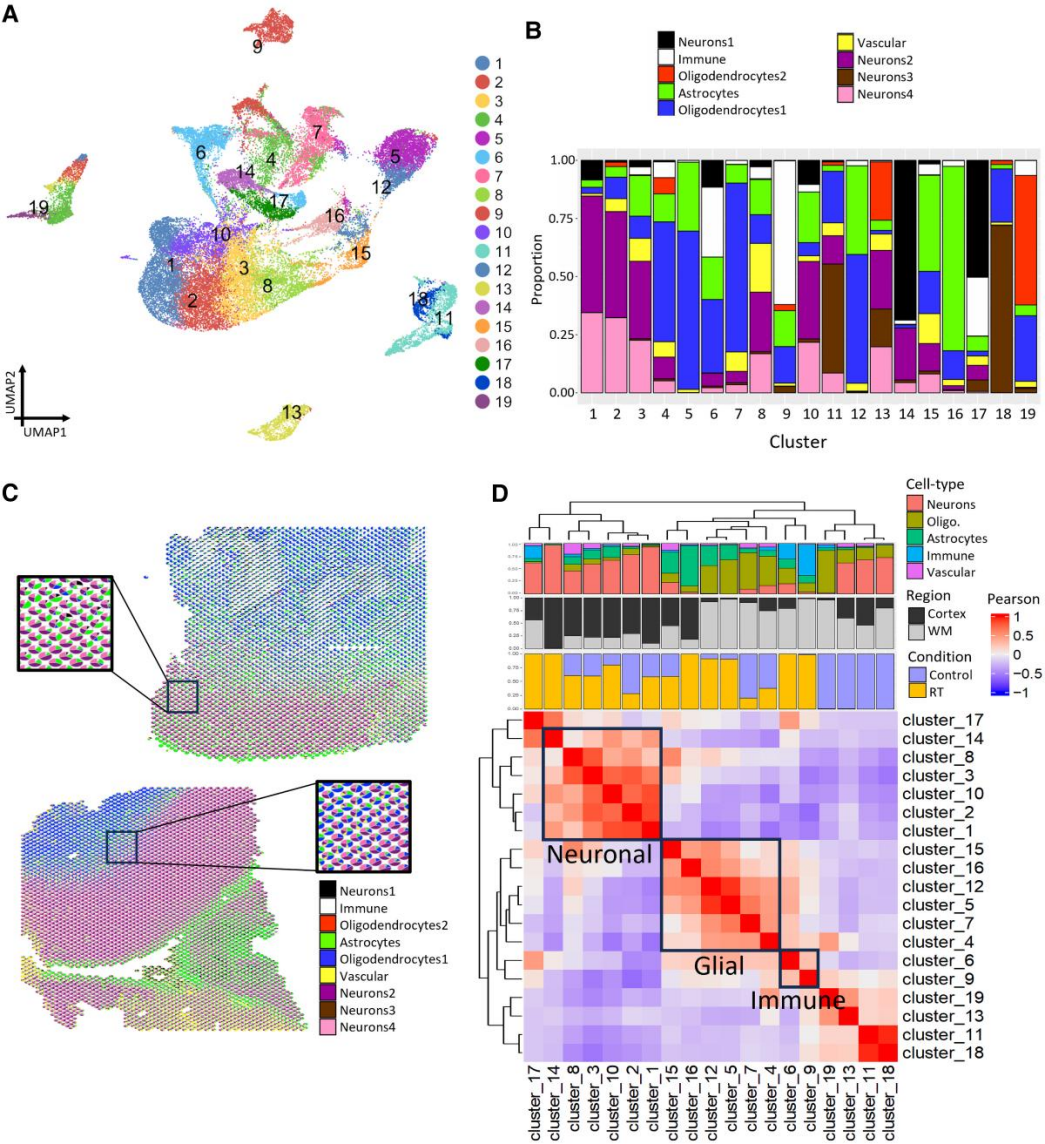
**Spatial transcriptomics reveals specific micro-environmental niches after irradiation**. (**A**) UMAP plot showing all spatial transcriptomic (ST) spots after filtering (39 157 in total). Each dot corresponds to a single ST spot, coloured by cluster, after unsupervised clustering. (**B**) Bar plot depicting the proportion of deconvoluted cell types in each cluster using STDeconvolve. (**C**) Two representative spatial plots showing deconvolved cell-type distribution across the sample. Each pie chart represents a ST spot and the proportions of the pie chart represent the deconvolved cell-type proportions (see also Supplementary Fig. 3). Colours are as for **B**. (**D**) Clustered correlation plot for ST cluster marker genes and bar plots of the proportion of cortex/white matter location, experimental group and cell-type proportions for spots within each cluster. Groups of clusters are annotated as per the main text. UMAP = uniform manifold approximation and projection.

After filtering, 39 151 spots were included in the final analysis, which identified 19 clusters upon unsupervised clustering (Fig. 2A and Supplementary Fig. 3A). Since the Visium ST spots were 55 µm-wide, each cluster represented a micro-environmental niche rather than a single cell. Therefore, we used STDeconvolve to computationally determine the cell-type composition of the clusters. This method effectively recovers cell-type transcriptional profiles and proportions within the ST data without relying on external single-cell references.^16-18^ Each deconvolved cell-type was annotated using a combination of cluster markers (Supplementary Fig. 3B) and most highly expressed genes, compared with expression in known cell types, using the Human Protein Atlas and STAB database,^19^ as well as the spatial location of cell types (Fig. 2C, Supplementary Fig. 3A and Supplementary material). Annotations were supported by correlation with known lineage markers (Supplementary Fig. 4A) and by comparison with cell-type signatures from published data^20^ (Supplementary Fig. 4B). We annotated four neuronal subtypes: Neurons1 (*VGF*, *NPTXR*, *DCLK1*); Neurons2 (*ATRNL1*, *VSNL1*, *STMN2*); Neurons3 (*SCG2*, *HSP90AB1*, *CALR*); and Neurons4 (*CAMK2A*, *NCDN*, *SNAP25*). These subtypes did not display distinct markers of well-established neuronal subtypes and were pan-cortical; therefore, we were not able to annotate more specifically. We annotated three glial cell-types: astrocytes (*GFAP*, *CLU*, *AQP4*); oligodendrocytes1 (*MOG*, *MAG*, *PLP1*); oligodendrocytes2 (*SCD*, *PPDPF*, *HSP90AA1*); and one vascular cell-type (*PTN*, *HBA1*, *ATP1A2*, *APOD*). Finally, there was one cell type that displayed a mixture of markers of both microglia/macrophages (*CD163*, *C1QB*, *NPC2*, *LAPTM5*) and leukocytes (*IGLC1*, *IGLG1*, *IGKC*) and therefore was annotated as a broad immune cell-type.

To further assess similarities between clusters, we computed cluster gene specificity scores for all marker genes and performed Pearson correlation between signatures, which showed clusters with similar cell-type compositions to be closely correlated (Fig. 2D). These combined data were then used to annotate micro-environmental niches.

Clusters 1–3, 8, 10, 14 and 17 were made up predominantly of neurons and labelled as neuronal niches. There was a varying contribution from control and RT samples, except Clusters 14 and 17, which were almost exclusively from RT samples. Within these neuronal niches, there were also varying smaller numbers of astrocytes, oligodendrocytes and vascular cells. Pathway analysis of the most highly expressed marker genes for these clusters showed pathways heavily weighted towards neuronal signalling and synapse organization (Supplementary Fig. 5A and Supplementary material). Cluster 17 alone had predominantly enriched pathways related to inflammation (Supplementary material), consistent with its significant immune component.

Clusters 4, 5, 7, 12, 15 and 16 were predominantly composed of oligodendrocytes and astrocytes, with varying smaller contributions of other cell types. Within this labelled glial grouping, Clusters 4 and 7 were enriched in control spots, whilst 5, 12 and 16 were enriched in irradiated spots (Fig. 2D). Clusters 4, 5, 7 and 12 were oligodendrocyte predominant. Astrocytes formed the most numerous cell type in Clusters 15 and 16, more marked in Cluster 16, whilst Cluster 15 contained a significant proportion of other cells. Pathway analysis for Clusters 4, 5, 7 and 12 showed pathways involved in gliogenesis, oligodendrocyte function and some inflammatory pathways (Supplementary Fig. 5A and Supplementary material), whilst pathway analysis for Clusters 15 and 16 highlighted pathways involved in regulation of the extracellular matrix (ECM), inflammation and developmental pathways for Cluster 16 (Supplementary material).

Cluster 9 was predominantly composed of immune cells, with a smaller contribution from glial cells, whilst Cluster 6 had a similar proportion of immune cells and oligodendrocytes and a smaller proportion of astrocytes (Fig. 2D). Gene ontology (GO) analysis using cluster makers for these labelled immune active clusters highlighted pathways that were heavily enriched for those involved in inflammatory processes, reflecting wide inflammatory responses (Supplementary Fig. 5A and Supplementary material). Marker genes for both clusters included many cytokines and chemokines, and other components of inflammatory cascades (Supplementary material). These clusters may reflect the direct effectors of neuroinflammation after radiotherapy.

Clusters 13 and 19 were closely correlated and from control samples, but while Cluster 13 was predominantly comprised of neurons with a smaller contribution of other cells, Cluster 19 was predominantly oligodendrocytes. Pathway analysis of marker genes for these clusters showed non-specific developmental pathways (Supplementary material). Clusters 11 and 18 were highly correlated, consisted predominantly of neurons with a smaller oligodendrocyte component and were both made up of spots from a single control sample (Cnt2; Supplementary Fig. 3A, D and E), whilst pathway analysis of marker genes from these clusters again showed non-specific developmental pathways (Supplementary material). These four clusters were not included in downstream analyses, as it was felt any findings would be difficult to generalize given the above features.

In conclusion, ST data from our patient cohort enabled clustering and annotation of micro-environmental niches, allowing identification of predominant neuronal and glial niches impacted by irradiation, as well as niches only seen in irradiated brain, with prominent inflammatory cells.

### Glial niches show inflammation, vascular and ECM changes—neurons secrete pro-inflammatory neuropeptides

**Figure 3:**
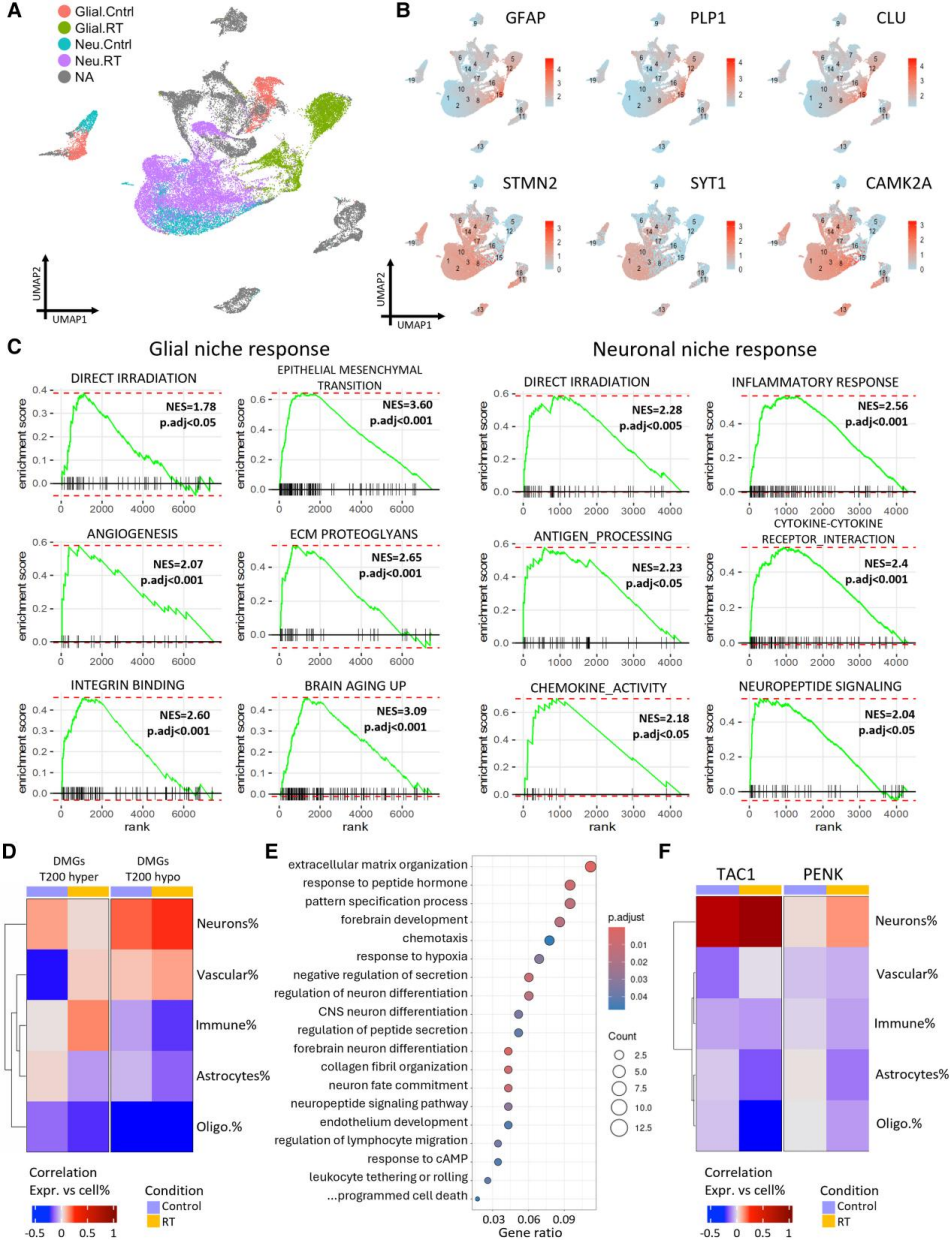
**Irradiated neurons residing in an inflammatory micro-environment with active neuropeptide and cytokine signalling**. (**A**) UMAP plot showing all spatial transcriptomic (ST) spots, coloured by control and radiotherapy (RT) glial and neuronal clusters. (**B**) UMAPs of all ST spots showing expression of glial (GFAP, PLP, CLU) and neuronal (STMN2, SYT1, CAMK2A) markers. (**C**) Selected Gene Set Enrichment Analysis plots for differentially expressed genes from RT glial clusters (*left*) and RT neuronal clusters (*right*). (**D**) Heat map of correlation between signature score for top 200 hyper- and hypomethylated genes from bulk DNA methylation and cell-type proportions of all ST spots. (**E**) Selected gene ontology pathways from 124 genes concordantly upregulated in irradiated neuronal niches (see also Supplementary Fig. 5E). (**F**) Heat map shows correlation between TAC1 and PENK expression and cell-type proportions of all ST spots. DMG = differentially methylated gene; NES = normalized enrichment score; UMAP = uniform manifold approximation and projection.

First, within glial predominant clusters, we directly compared ST spots from samples that had undergone RT to those which had not (Fig. 3A and B). GSEA of differentially expressed genes (DEGs) (Supplementary Fig. 5B) revealed the pathways included many related to changes in the ECM, inflammation and angiogenesis, as well as developmental pathways (Fig. 3C and Supplementary material). These findings are consistent with the current mechanistic understanding of brain injury after radiation, in which glial and immune cells act as effectors of neuroinflammation, likely in response to secreted factors from neurons and other cell types and disruption of the neurovasculature, and these changes lead to the chronic injury seen in patients. Changes to the ECM after radiation have been predicted from *in vitro* and mouse models,^21^ but these have not been confirmed in human tissue outside of the glioblastoma literature, which is likely specific to that disease setting, given its diffusely infiltrative nature. In our data, we saw the upregulation of many collagen genes (*COL8A1*, *COL3A1*, *COL6A3*, *COL5A1*, *COL1A2*, *PCOLCE*) as well as *TNC*—a glycoprotein highly expressed in the ECM during development and in the adult CNS.

Whilst glial and immune cells are thought to be the direct effectors of neuroinflammation post-RT, the inflammatory cascade is suspected to be driven to a large part by the response to neuronal damage, which then contributes to activation and senescence of astrocytes, microglia and endothelial cells.^4,5^ The damaged neurons secrete pro-inflammatory factors, which are currently not fully identified; hence, we next focused in more detail on irradiated neuronal niches.

Next, we directly compared ST spots from samples that had undergone RT to those which had not within neuronal predominant clusters (Fig. 3A and B). Cluster 17 was not included as it had a prominent immune component and was felt to be distinct from other neuronal clusters. As an initial comparison between the irradiated and non-irradiated neuronal clusters, all spots within these clusters were scored against signatures from published datasets for broad biological responses. The irradiated neuronal clusters scored more highly for signatures of senescence^22^ and inflammation.^23^ Conversely, non-irradiated neuronal clusters scored more highly for an intact neuronal signature, previously found to be inversely associated with neurodegeneration in multiple sclerosis,^24^ which shares several features with radiation-induced injury (including chronic inflammation, increased reactive oxygen species, white matter pathology and long-term cognitive decline; Supplementary Fig. 5C). We then assessed clusters against specific elements of the inflammatory cascade (immune check-points,^25^ cytokine response,^26^ HLA association^27^ and inflammasome activation^26^) and found that irradiated neuronal clusters scored more highly for each signature (Supplementary Fig. 5D).

GSEA of DEGs (Supplementary Fig. 5B) between these irradiated and non-irradiated neuronal groups showed enrichment for pathways including direct irradiation, several inflammatory pathways and neuropeptide signalling (Fig. 3C). Closer examination of the neuropeptide signalling pathway identified several highly expressed neuropeptides and precursors (*TAC1*, *TAC3*, *PNOC*, *PENK*, *PDYN*, *GAL*, *NPW*, *CARTPT*) in the irradiated neuronal clusters. Of these, tachykinins *TAC1* and *TAC3*, and opioid precursors *PENK*, *PDYN* and *PNOC*, have known associations with components of the neuroinflammatory cascade (e.g. through CXCL8, IL6 and CCL2^28-30^) but have not previously been associated with radiation-induced brain injury.

Next, we assessed the correlation between cell types and an RT-methylation signature consisting of the 200 genes most hyper- and hypomethylated at promoters/5′UTRs/exons. We observed that the hypo-RT-methylation signature correlated most highly with neurons from irradiated samples (Fig. 3D), suggesting that changes to DNAme post-RT are not uniform across cell types and that neurons may be preferentially susceptible to disruption. When we integrated DNAme and expression data from irradiated versus non-irradiated neuronal niches, we found 124 genes that were concordantly upregulated in irradiated neuronal niches (Supplementary Fig. 5E). Pathway analysis of these genes showed that enriched pathways included inflammatory and neuronal differentiation pathways as well as neuropeptide signalling (Fig. 3E). Concordant genes involved in neuropeptide signalling included *TAC1* and *PENK*, highly suggestive that DNAme changes at these genes are important and sustained post-irradiation. Additionally, expression of *TAC1* and *PENK* was highly correlated with neurons in our ST data and was highest in neurons from irradiated samples (Fig. 3F and Supplementary Fig. 5F), validating their neuronal specificity.

We next analysed the expression of ∼3000 receptor-ligand (R-L) pairs from CellPhoneDB v5^31,32^ in our ST data (Supplementary Fig. 6A and Supplementary material, ‘Materials and methods’ section) to examine the interactions that control disease mechanisms directly within tissue.^24,33^ Interactions within clusters predominated over interactions between clusters in all samples (Supplementary Fig. 6B). It should be noted here that since spots of the same cluster are usually adjacent, the potential interaction within clusters outweighs that for interactions between clusters; thus, communication within the milieu of each niche will likely reveal important interaction patterns, especially considering that whilst each spot in our comparisons is predominantly neuronal or glial, there are other admixed cell types. The most substantial interactions were within and between the groups of predominantly neuronal Clusters 1–3 and 10 in both control and RT samples, but smaller numbers of interactions were seen between most other clusters (Supplementary Fig. 6B). We then calculated R-Ls that had significantly different interaction between irradiated and non-irradiated spots and categorized these interactions by the direction of signalling.

**Figure 4:**
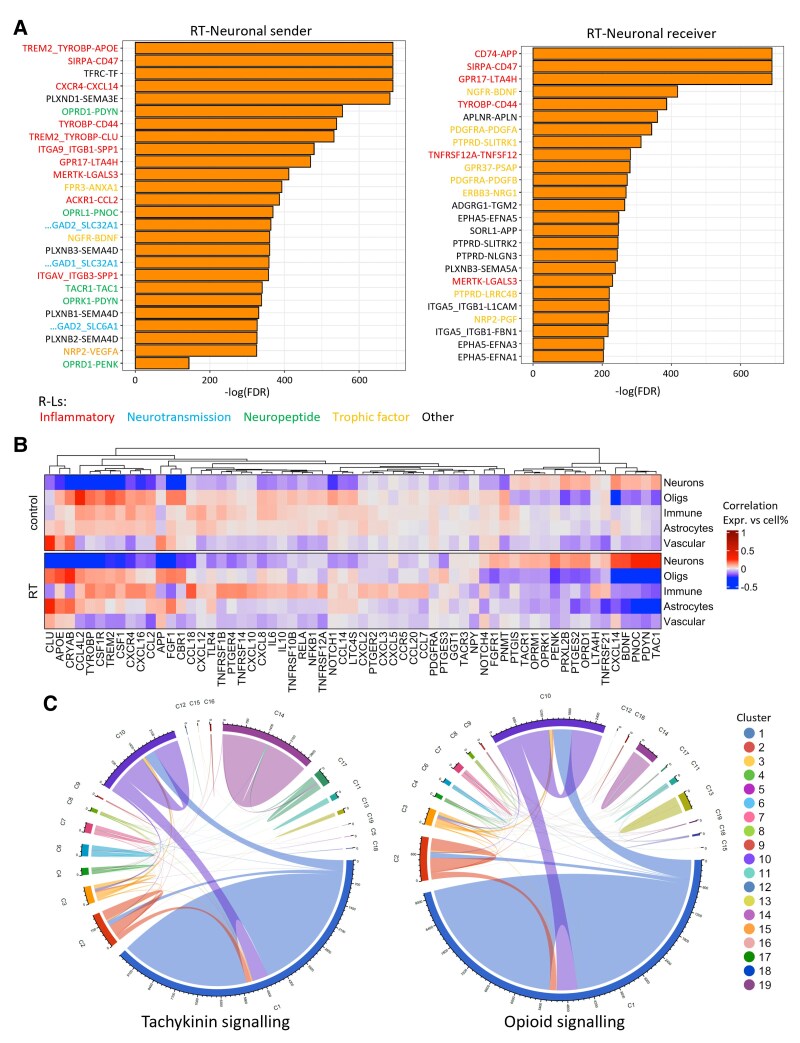
**Receptor-ligand analysis of spatial transcriptomic data confirms neuropeptide activity**. (**A**) Bar plots showing the 25 most significantly different receptor-ligand (R-L) pairs when interaction scores were compared between control and radiotherapy (RT) neuronal clusters. Additionally, OPRD1-PENK is shown in the RT-neuronal sender plot; see also Supplementary material. Interaction scores between clusters were compared using a Wilcoxon test, and *P*-values were adjusted for multiple hypothesis testing using the Benjamini-Hochberg procedure. (**B**) Heat map dendrogram showing correlation between expression of genes from R-L analysis with cell type proportions in all spatial transcriptomic (ST) spots. (**C**) Circos plots showing tachykinin and opioid R-L interactions between ST clusters. Chord thickness represents the number of R-L interactions. FDR = false discovery rate.

Examining the most significant R-L interactions sent from RT-neuronal spots, inflammatory signalling was prominent, and there were several active neuropeptide interactions, including TAC1 and opioid signalling (Fig. 4A), supportive of *TAC1* and *PENK* activity having functional consequences within neuronal niches post-RT. Additionally, neurotransmitter R-Ls involved in GABAergic signalling/uptake were present, as well as semaphorin signalling (involved in axon/neuron plasticity). Within the most significant R-L interactions where RT-neuronal niches received signals, there was a mixture of inflammatory, trophic and other signals. The trophic factors included BDNF and PDGFA, both known to be involved in maintaining neurons after injury. Several Ephrin pairs were also identified (diverse functions, including synapse maintenance). When we examined the most highly interacting R-L senders in the glial niches, there was more balanced inflammatory and trophic signalling (Supplementary Fig. 6C). CLU and APOE signalling were seen, and both these pathways are involved in astrocyte response to injury. These genes were also seen in the neuronal niche senders, likely from reactive astrocytes in close proximity to neurons. Trophic factors here included FGF and PDGFRA. The signals received by the glial niches were from neurotransmitters involved in glutamate signalling and calsyntenin (cadherin family, calcium-binding, transmembrane proteins).

Correlation of selected genes from this R-L analysis with cell-type proportions within ST data (Fig. 4B) revealed that the identified neuropeptide precursors were most highly correlated with neurons in RT samples, as were the tachykinin and opioid receptors. Genes with known roles in glial responses to injury (*CLU*, *APOE*, *CRYAB*) were most highly correlated with glial cells in RT samples. There were also inflammatory mediators expressed by immune cells alone, likely microglia/macrophages (*CCL18*, *TNFRSF1B*, *CXCL10*), as well as those expressed more generally by astrocytes, oligodendrocytes and immune cells (*TYROBP*, *CCL4L2*, *CSF1R*). These findings were consistent with the hypothesis that neuroinflammation seen post-RT is directly mediated by glia and immune cells through cytokines and other inflammatory molecules, and that neuropeptides released from irradiated neurons could drive this inflammation. Both tachykinin and opioid signalling took place mainly between neuronal Clusters 1, 2 and 10 (Fig. 4C), reflecting the dual roles of tachykinins and opioids as neurotransmitters and immunomodulators, indicating that these clusters have more overall interactions and revealing that adjacent spots are more likely to harbour interactions.

This characterization of RT-glial niches with ST supports roles for chronic inflammation and disruption of brain microvasculature and ECM post-irradiation. The direct comparison between irradiated and non-irradiated neuronal niches showed that irradiated neurons reside in a highly inflammatory micro-environment that may be actively maintained by a combination of neuropeptide and cytokine signalling. DNAme appears to be involved in the regulation of some of these genes, and neurons may be more susceptible than other cells to these changes.

### DNA methylation disruption in irradiated CO model is similar to that in patient tissue

To further understand the contribution of DNAme changes to the phenotype induced by irradiation, we established a CO model that enables assessment at early time points post-irradiation, which is not possible in patient material. COs were irradiated at Day 48 of development with a patient-matched dose (24 Gy). The time point was chosen to enable the impact of irradiation on the early neuronal response to be studied in a complex cellular environment harbouring neurons that display markers of maturity (NeuN, TBR; Supplementary Fig. 7A–C), which are present within neuropil areas between rosettes, where there is a relatively limited non-neuronal population.^34,35^

**Figure 5:**
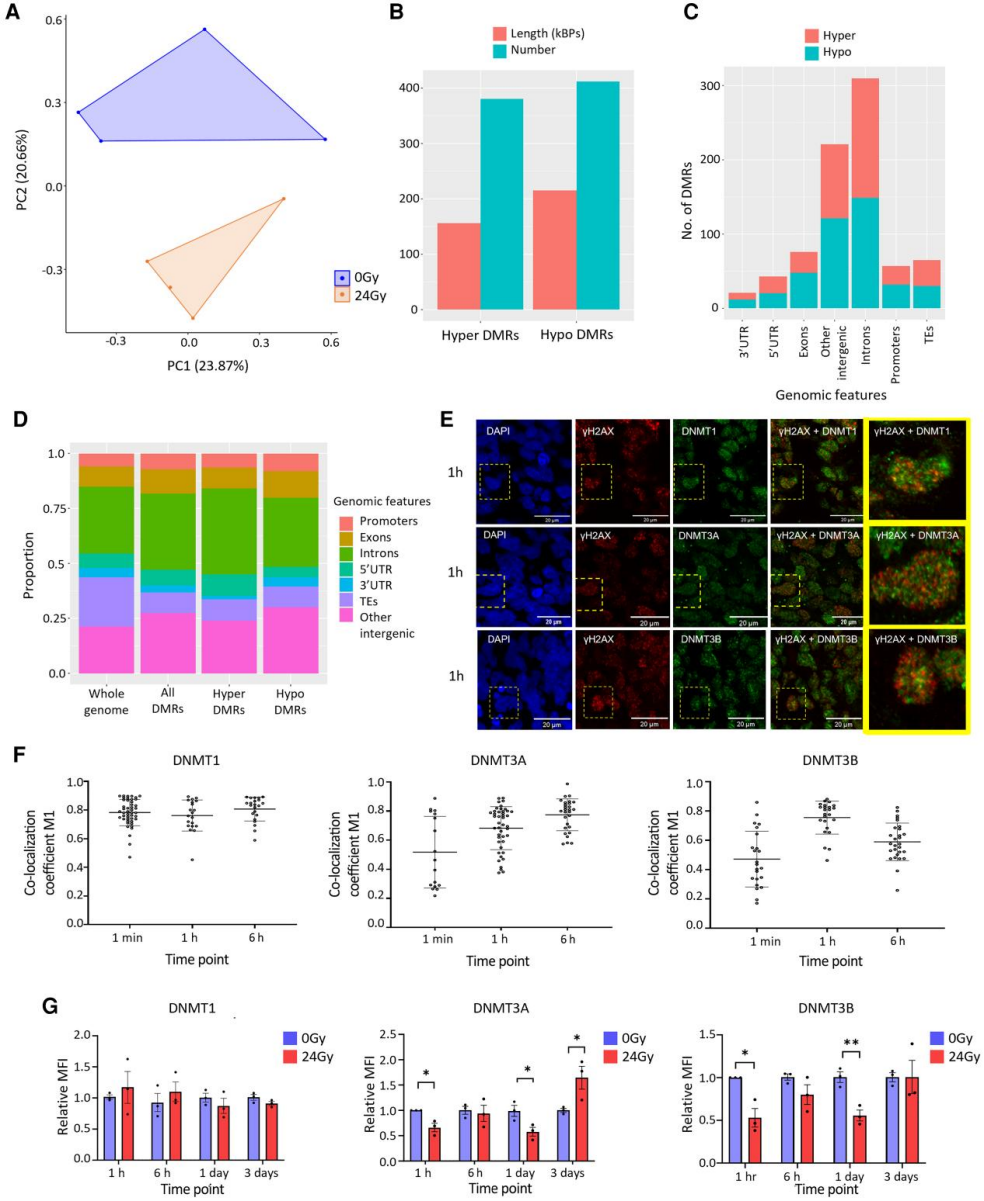
**After irradiation in a cerebral organoid model, disruption of DNA methylation patterns is accompanied by dysregulation of DNMTs**. (**A**) Principal component analysis of cerebral organoid (CO) DNA methylation data using all probes after standard filtering. (**B**) Bar plot showing number and length (in kilobase pairs) of hyper- and hypomethylated DMRs. (**C**) Bar plot depicting the number of DMRs by genomic region, with the proportion of hypermethylated (red) and hypomethylated (blue) DMRs. (**D**) Bar plot showing genomic location of DMRs as a percentage. The *left* bar is the whole genome as represented on the DNA methylation array. (**E**) Representative immunofluorescence images illustrating colocalization of γH2AX (red) with DNMT1, DNMT3A and DNMT3B (green) at 1 h post-irradiation. The overlay images demonstrate points of colocalization in yellow. Nuclei were counterstained for DAPI (blue). Scale bars are 20 μm. See also Supplementary Fig. 9B–D. (**F**) Dot plots of Manders colocalization coefficient (M1) value for γH2AX colocalizing with DNMT1 (*left*), DNMT3A (*centre*) and DNMT3B (*right*) from individual nuclei from COs at time points ranging from 1 min to 6 h post-irradiation. The error bars represent standard deviation. See also Supplementary Fig. 9B–D. (**G**) Bar plots depicting relative mean fluorescence intensity (MFI) of DMNT1 (*left*), DNMT3A (*middle*) and DNMT3B (*right*) at time points ranging from 1 h to 3 days post-irradiation. Each data-point on the graph represents pooled data from a single CO. Unpaired *t*-test was performed to assess statistical significance. Error bars represent the standard error of the mean. See also Supplementary Fig. 10. *P ≤ 0.05. DMR = differentially methylated region; TE = transposable element.

Firstly, DNAme changes were characterized in COs 4 weeks post-irradiation to assess the extent to which findings would recapitulate results from patient samples. A PCA of all DNAme probes showed distinct clustering between the groups (Fig. 5A). Annotation of DMRs showed that 52% were hypomethylated, demonstrating a more even representation of hypo/hyper-methylation compared to patient samples (Fig. 5B), which had shown strong hypomethylation bias. As with the patient samples, the ratio of hypo- to hypermethylated DMRs in genomic regions was reflective of the overall distribution of DMRs, showing mildly increased hypomethylation within promoters (64%), exons (64%), introns (53%), TEs (57%) and other intergenic regions (63%), whereas 3′UTRs were predominantly hypomethylated (79%) and 5′UTRs were mildly hypermethylated (60%) (Fig. 5C). In COs, the overall enrichment patterns of DMRs at genomic locations were very similar to that of the patient material (Fig. 5D, Supplementary Fig. 8A and Supplementary material); however, the effect size of the changes was lower, with only the depletion of DMRs from TEs and enrichment of hypermethylated DMRs in introns, as well as DMRs overall reaching those equivalent to patient data.

We then mapped the CO DMRs to DMGs, and when we compared DMGs from patients to our CO model, 30% (203/672) of all CO DMGs were shared with patients (Supplementary Fig. 8B). The GO pathway analysis highlighted terms related to development and neuronal regulation, synapse formation and brain development more generally (Supplementary Fig. 8C). Many transcription factors were present in the shared DMGs, including *SOX1* and *PAX6*, suggesting that changes to specific genes with far reaching downstream consequences were shared in the two settings. *CLU* and *CRYAB* were also among the shared genes.

Next, we set out to leverage our CO model, which shares some similarities in the dysregulation of DNAme post-irradiation with patient samples, to explore whether known mechanisms of DNAme were altered. First, we assessed whether the model recapitulated known downstream cascades. DNA breaks caused by radiation occur through direct particle damage and indirect damage through the generation of ROS, with the NF-kB pathway being a common effector after stress and DNA damage.^36^ We showed increased oxidative DNA damage post-irradiation in our model, as assessed by DNA damage markers, up to 6 h post-irradiation, resolving at 1 day, consistent with established time frames^37^ (Supplementary Fig. 8D-E and Supplementary Fig. 9B-D). However, no evidence of the nuclear translocation of NF-kB (p65), as evidence of pathway activation, was detected (Supplementary Fig. 9A).

Whilst mechanisms have been proposed for how radiation could disrupt DNAme, the process by which this happens at clinically relevant doses has not been studied. The relationship between radiation and DNAme alteration is complex but thought to involve direct effects on the methylome by reactive oxygen species and dysregulation of enzymes that methylate or demethylate cytosines,^7,8,38^ including the maintenance DNA methyltransferase DNMT1, the *de novo* methyltransferases DNMT3A and DNMT3B and TET demethylases. There is evidence that DNMTs are involved in repairing DNA methylation patterns during the DNA damage response.^39-41^ Indeed, irradiation of COs revealed colocalization between the DNA damage marker γH2AX and DNMTs (Fig. 5E and F). DNMT1 was colocalized at all time points when DNA damage was observed (Supplementary Fig. 9B). For DNMT3A, this was most pronounced at 6 h (Supplementary Fig. 9C) and for DNMT3B, at 1 h post-irradiation (Supplementary Fig. 9D). We then assessed the levels of DNMTs with immunofluorescence in COs at time points up to 3 days post-irradiation and found decreases in DNMT3A and 3B at 1 h and 1 day, with a subsequent increase in DNMT3A at 3 days, whilst DNMT1 was unchanged over the same period (Fig. 5G and Supplementary Fig. 10).

In conclusion, our CO model of irradiation harboured DNAme changes with similarities to those observed in patient samples, although less marked. This model allowed us to gain insights into potential mechanisms, including the dysregulation of key effectors of the DNAme machinery, which may contribute to the observed epigenetic changes post-irradiation.

### Irradiated neuronal phenotype of patient tissue recapitulated in the CO model at early time points

**Figure 6:**
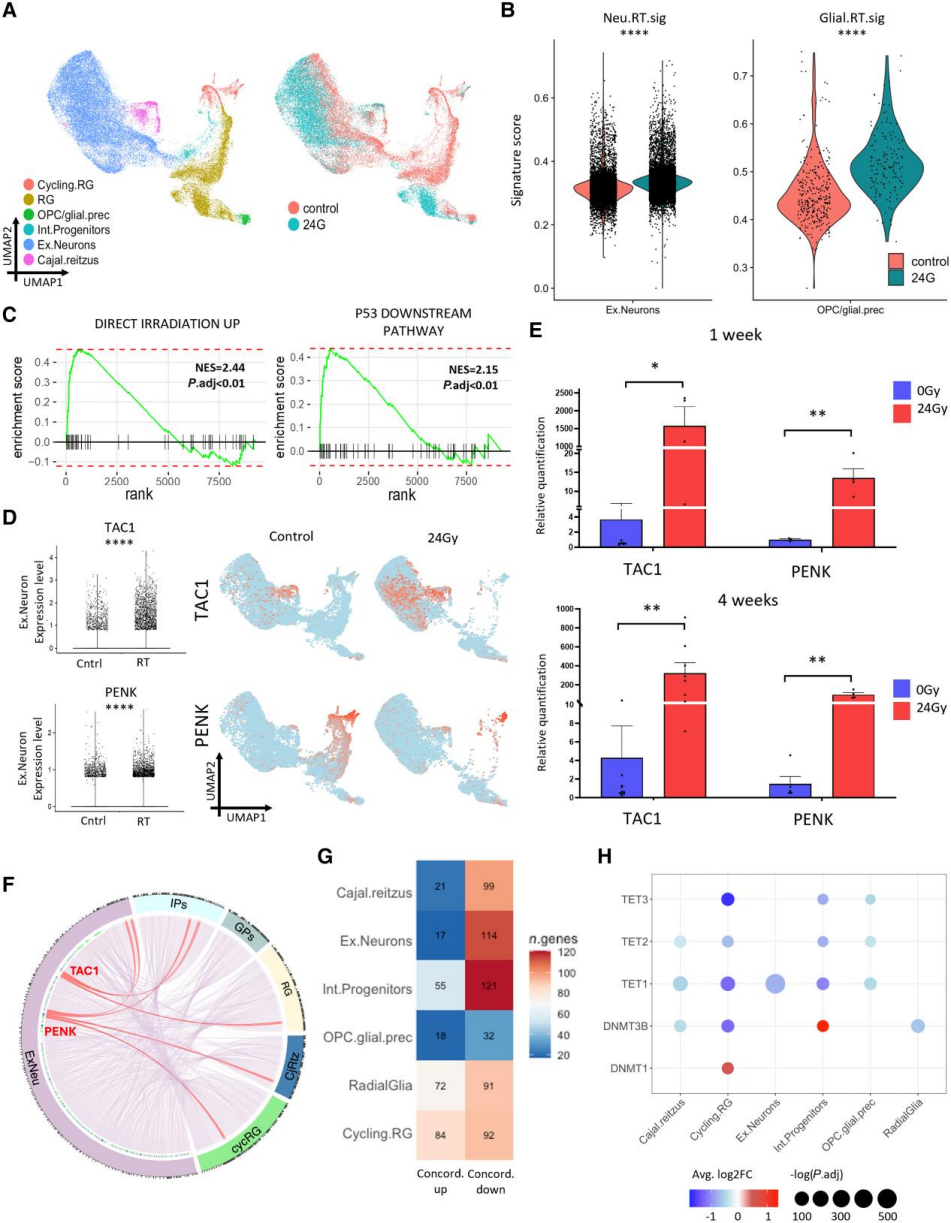
**Single-cell RNA sequencing in cerebral organoid model shows recapitulation of irradiated neuronal phenotype at early time points**. (**A**) UMAP plot showing neuroglial cells, coloured by cell-type (*left*) and experimental condition (*right*). (**B**) Violin plots for signature scores of spatial transcriptomic (ST) neuronal radiation signature in excitatory neurons *(left*) and for ST glial radiation signature in oligodendrocyte/glial precursors (*right*). Wilcoxon test. (**C**) Selected Gene Set Enrichment Analysis plots for all excitatory neuron differentially expressed genes. (**D**) Expression of *TAC1* (*top*) and *PENK* (*bottom*) in excitatory neurons is shown in the box and whisker plot (*left*) and UMAP with all neuroglial cells (*right*). Wilcoxon test. (**E**) Bar plots depicting relative expression of TAC1 and PENK using RT-qPCR at 1 and 4 weeks after irradiation. Significance was tested with unpaired *t*-test. (**F**) Circos plot showing all significant receptor-ligand interactions involving ex.Neurons. *TAC1* and *PENK* interactions are highlighted in red. (**G**) Heat map shows concordant genes in the cerebral organoid (CO). (**H**) Bubble plot shows changes in DNA methylation machinery genes after irradiation by cell type. *P ≤ 0.05, **P ≤ 0.01, ***P ≤ 0.001, ****P ≤ 0.0001. Ex.Neuron = excitatory neuron; NES = normalized enrichment score; OPC = oligodendrocyte progenitor cell; RG = radial glia; RT = radiotherapy; UMAP = uniform manifold approximation and projection.

We performed single-cell RNAseq (scRNAseq) on COs collected 24 h post-irradiation, enabling early transcriptional responses of neurons to be modelled at a time point not available in patient material. We performed Louvain clustering (Supplementary Fig. 11A) and annotated cell types based on marker gene expression (Supplementary Fig. 11B) and GO pathway enrichment (Supplementary Fig. 11C), comparing these against a large, published reference dataset^42^ (Supplementary Fig. 11D). We used COs from two independent patient-derived expanded potential stem cell (EPSC) lines and found that the cell-type composition between the two lines varied, with one containing a greater proportion of cortical hem and mesenchymal progenitors and the other comprising a higher proportion of neuroglial cells (Supplementary Fig. 11E and F). This variability is not uncommon to encounter in COs, and therefore we next re-clustered the data, retaining only neuroglial cell types for further analysis, as those most relevant to this study (Fig. 6A): radial glia (*SOX2*, *FABP7*, *SFRP1*); glial precursors (*S100B*, *PLP1*, *SOX10*); intermediate progenitors (*HES6*, *NEUROG2*); excitatory neurons (*STMN2*, *NEUROD2*, *DCX*, *NEUROD6*); and Cajal-Retzius cells (*SCG2*, *RELN*; Supplementary Fig. 11B). Of these, excitatory neurons are most similar to mature neurons found in the human adult brain.

Cells in different cell cycle phases displayed differential radiosensitivity, reflected by the increase in G1 cells and decrease in G2M cells 24 h post-irradiation (Supplementary Fig. 11G). Correspondingly, there were changes in cell-type proportions, with the more proliferative cell types most decreased post-irradiation (Supplementary Fig. 11H).

To assess the similarity between irradiated brain and the irradiated CO model, patient-derived irradiation signatures were compiled from the top 75 upregulated genes from the neuronal and glial DEGs from ST data (Supplementary Fig. 5B). CO neurons and glial precursors were scored against these patient-derived signatures, and the irradiated neurons and glial cells indeed scored more highly (Fig. 6B). We then performed differential expression analysis (Supplementary Fig. 11I) and GSEA of DEGs from excitatory neurons highlighted pathways, including direct irradiation and p53 signalling (Fig. 6C), whilst GO enrichment showed the involvement of pathways including DNA damage via p53, apoptosis, DNA damage check-points, neurotransmitter biosynthesis and regulation of cytokines (Supplementary Fig. 11J).

We saw an inflammatory phenotype at this very early stage post-irradiation, as well as pathways involved in DNA damage, which was expected given the proximity of the radiation dose. After assessing whether the two neuropeptide precursors we had identified in human tissue were similarly upregulated, we found that the expression of both was increased in excitatory neurons (Fig. 6D). Furthermore, this upregulation persisted at 1- and 4-weeks post-irradiation seen by qPCR performed on whole organoids (Fig. 6E). Additionally, when significant R-L interactions involving ligands expressed by neurons were examined, neuropeptides produced by *TAC1* and *PENK* were predicted to be involved in signalling between neuroglial cells at early time points post-irradiation (Fig. 6F). Additionally, in the irradiated glial precursor cells in the CO model, several genes involved in inflammation from our ST data were also overexpressed as DEGs: *IL6*, *TNFRSF10B*, *TNFRSF1B*, *CCL7*, *CCL18*, *CXCL5* and *TYROBP* (Supplementary material). When we integrated DNAme and DEGs, we found that concordant genes had predominantly decreased expression in all cell types (Fig. 6G).

We also assessed changes in the expression of DNMTs and TETs in neuroglial cell types in the scRNAseq data (Fig. 6H). The most striking finding was widespread downregulation of TETs, with all cells except radial glia showing some TET depletion. Intriguingly, neurons showed a decrease in *TET1*, known to influence the neuronal methylome and gene expression. At the transcript level, we did not see significant changes in *DNMT3A/B* in the neurons observed in immunofluorescence experiments, but these changes may not be detectable at the transcript level in this setting. *DNMT3B* expression was decreased in both radial glia cell types and Cajal-Retzius cells but increased in intermediate progenitors, and *DNMT1* was increased in cycling radial glia. These changes suggested cell-type specific effects of radiation on DNMT levels, with more proliferative cells showing increases but a more widespread decrease in TETs at this time point.

In summary, in a CO model of irradiation at early time points, we saw that neurons comparable to those seen in patient tissue showed an inflammatory phenotype in response to DNA damage, which was established by 24 h post-irradiation. Expression of *TAC* and *PENK*, found to be overexpressed in neurons post-RT in patient tissue, was also increased in the CO model at 24 h, as well as at 11- and 4-weeks post-irradiation, suggesting that they are upregulated as an early response, before any downstream chronic neuroinflammation has developed. We also saw changes in the levels of DNMTs and TETs at 24 h post-irradiation that appeared to be cell-type specific.

## Discussion

**Figure 7:**
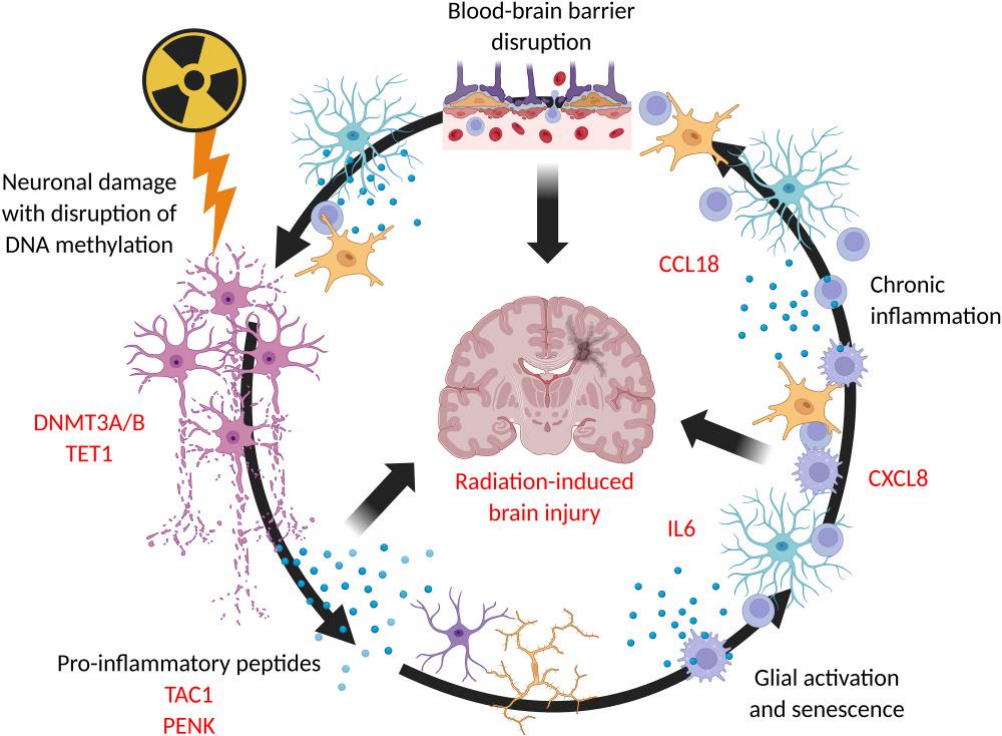
**Schematic showing proposed interaction between targeted radiotherapy, disruption of DNA methylation and chronic neuroinflammation to produce radiation-induced brain injury**. See main text for details; Created in BioRender. Marino, S. (2025) https://BioRender.com/w6bjgma.

We identified distinct differences in DNAme patterns in peri-lesional brain tissue after targeted-RT, characterized micro-environmental niches post-irradiation and identified neuropeptides known to play a role in neuroinflammation within irradiated neuronal niches. We modelled early radiation changes in a CO system, where we focused on neurons, and showed partial overlap of DNAme changes with those seen in human tissue samples, as well as dysregulation of the expression of neuropeptide precursors, which could play a role in initiating radiation-induced neurotoxicity. Finally, we established that specific elements of the DNAme machinery, including DNMTs and TETs, with roles in DNA damage repair, are likely dysregulated after irradiation in a cell-type specific context (Fig. 7).

Alteration of DNAme is involved in the pathogenesis and progression of many neurological disorders, including neurodegeneration,^43^ multiple sclerosis^44^ and stroke.^45^ Radiation can also disrupt DNAme patterns; however, the findings are highly variable, depending on the type and dose of radiation and the experimental system. The existing evidence for these alterations is derived almost entirely from neoplastic cells and non-CNS mouse tissues, with no investigations in clinically relevant human brain tissue. We have shown distinct DNAme changes in irradiated human tissue, including a bias towards hypomethylation of DMRs. The genomic location of these DMRs, which were preferentially found at promoter, exon and 5′UTR regions, predicts an influence on gene expression and potentially a strong phenotypic impact.

The time between RT and subsequent resection was variable in our cohort (from 7–240 months). Whilst this enabled changes in a clinically relevant time frame to be studied, i.e. capturing the long-term sequelae of RT, it was not suitable for discriminating between immediate and long-term changes. RT is a potent inducer of DNA damage, and it has been shown that stable changes in DNAme after DNA damage occur within a discrete early time frame^41^ and that this early disruption can persist as epigenetic ‘scars’.^41,46,47^ Supporting this, several animal models have shown disruption of DNAme that persists for months after radiation exposure,^48-51^ whilst many of the side effects of RT are observed up to years after treatment, suggesting a possible link between DNAme alterations and radiation-induced late side effects. To unpick those alterations in human brain tissue samples potentially directly linked to radiation, we set up a CO model of RT. We reasoned that since the common consensus is that neuronal damage is a major driver of RT-induced neuroinflammation, this model, which captures neuronal development well, would be suitable to identify early changes leading to these epigenetic scars. Indeed, when we examined the DNA methylome at early time points post-irradiation in COs, we again found distinct patterns of DNAme, with DMRs distributed similarly across the genome, as in the patient samples. Differences were also observed. For example, DMRs were less hypomethylated overall in COs compared to patient samples. This may have reflected the change in the expression of TETs at this early time point but possibly the diverse cellular composition of the samples; the changes, although similar, were less marked in the CO model. Arguably, we therefore captured irradiation-specific DNAme changes in our model.

Interestingly, when we compared DMGs in patient tissue samples and COs post-irradiation, we identified genes common to both settings, including those involved in neurogenesis, synapse formation, neuroinflammation and transcription factors. These genes may represent those particularly sensitive to the disruption of DNAme, both immediately after irradiation and over time. The neuronal response to DNA damage is complex, with repair not uniform across the genome but dependent on cell-specific transcriptional activation.^52,53^ There are multiple lines of evidence linking the reconstitution of DNAme patterns and DNA damage repair pathways.^40,54^ Whilst the exact mechanisms of DNAme reconstitution in this context are not well understood in human tissue, the process appears to be error-prone. These errors can lead to a revision of the methylome in repaired genes, producing ‘epi-alleles’ in individual cells that lead to differential gene expression.^41,46,55^

Our CO model revealed changes in the expression levels of DNMT3A and DNMT3B at early time points post-irradiation. These DNA methyltransferases are involved in DNA methylation reconstitution,^39-41,56^ and we showed their colocalization with DNA damage foci in our model. This colocalization occurred in the same time frame as the changes in DNMT levels were observed. Our scRNAseq data suggested that these changes are likely to be cell-type specific, with a more general reduction in TET levels across neuroglial cell types. TETs are demethylases that convert 5-mC to 5-hmC and have a complex relationship with DNA damage repair pathways,^57,58^ whilst TET-coupled demethylation is also involved in neuroprotection following neuronal damage.^59,60^ Specifically, TET1, shown to shape the neuronal methylome, affecting activity-related gene expression,^61-63^ was decreased in irradiated neurons in COs at 24 h. Alterations in the expression and cellular localization of these essential components of the DNAme machinery in response to irradiation, at time points where their functions are required for DNAme reconstitution after DNA damage, could therefore contribute to the changes in DNAme seen in patient samples after irradiation.

We have leveraged ST to characterize micro-environmental niches impacted by irradiation and identified neuropeptide precursors that are differentially methylated in patient tissue within irradiated neuronal niches. Upon the integration of bulk DNAme data with DEGs from neuronal niches in our ST data, we saw that *TAC1* and *PENK* are concordantly expressed/methylated. We also showed that *TAC1* and *PENK* expression is highly correlated with irradiated neurons, and the R-L interaction analysis supported their active signalling. These findings were set in the context of a marked inflammatory micro-environment. These genes were also overexpressed in irradiated neurons in the CO model, suggesting that they form part of an early response post-irradiation, before neuroinflammation has fully developed.

The *TAC1* gene encodes the tachykinin substance P (SP) and neurokinin A (NKA). Tachykinins are a family of evolutionarily preserved small neuropeptides^64^ that act as neurotransmitters and at the intersection of immune and nervous system communication.^64,65^ SP is known to be involved in neuroinflammatory responses to many insults, inducing vasodilation, promoting the recruitment of immune cells and augmenting the inflammatory responses of both infiltrating and resident cells.^28^ PENK and PDYN are precursors for the opioid peptides enkephalin and dynorphin, respectively. These are best known to act as neurotransmitters involved in pain processing. Additionally, opioids and their receptors are present on both glia and peripheral immune cells,^29,30^ and cross-talk occurs between opioid receptors and the chemokine and chemokine receptor families.^29^ Our results suggest that protracted overexpression of these neuropeptides could represent a key driver of post-irradiation neuroinflammation. There are drugs available that target tachykinin pathways, developed to reduce neuroinflammation,^28^ and their use as an adjuvant after RT could be trialled. Interestingly, the tachykinin receptor TACR1/NK-1R is overexpressed in some gliomas^66,67^ and contributes to glioma pathobiology, and there is evidence that this pathway is also involved in brain metastasis in breast cancer,^68^ suggesting that treatments modulating tachykinin function could be harnessed for a dual purpose, further increasing the benefits to patients with limited treatment options. Clinical modulation of opioid pathways is common. These routinely available drugs with clear immunomodulatory functions^69^ show promise for modulating neuroinflammation^70^ and could be utilized in the setting of radioprotection. Finally, there is mounting evidence that epigenetic dysregulation and neuroinflammation contribute to the pathogenesis of many common neurological disorders^43-45^; therefore, there may be scope for improving outcomes by manipulating the pathways we have uncovered.

One limitation of this study concerns the variability of patient samples, as they varied in terms of the origins of the tumours, time post-RT and location of brain lesions. This resulted from the challenges in compiling the unique biological resource used for this work. We aimed to statistically control for these factors while maximizing the sample size to increase statistical power. It must be noted that, for example, time post-irradiation may be worthwhile investigating in more detail and that confirmation of our findings in a larger cohort would offer the best validation of the results. Another limitation is that we were unable to identify suitable published data to allow for reference-based deconvolution with CO DNAme data, but we maintain that, given the notable parallels observed in phenotype and similarities seen in DNAme patterns, these data are informative and warrant additional exploration. Further work is also needed to address the full consequences of the observed changes in DNMT and TET levels on DNAme post-irradiation.

## Conclusions

We collected a novel cohort of patient tissue that had undergone targeted CNS RT and investigated this unique biological material with state-of-the-art molecular techniques. Our study links RT-induced neuroinflammation with the disruption of DNAme for the first time and highlights potential druggable mechanisms for this neuroinflammation.

## Supplementary Material

awaf163_Supplementary_Data

## Data Availability

All raw data is available on NIH GEO DataSets: DNA methylation: GSE264703; bulk RNAseq: GSE265797; spatial transcriptomics: GSE272334; scRNAseq data: GSE289218. All data contributing to the figures are available in the accompanying Supplementary material.
