# Supplementary material for: Disruption of DNA methylation underpins the neuroinflammation induced by targeted CNS radiotherapy
Source: Brain. 2025 Apr 29;148(9):3137–52. doi: 10.1093/brain/awaf163 (PMC12404709; doi:10.1093/brain/awaf163)
Supplement: awaf163_Supplementary_Data [file awaf163_supplementary_data.zip › brain-2024-01691-File010.pdf]

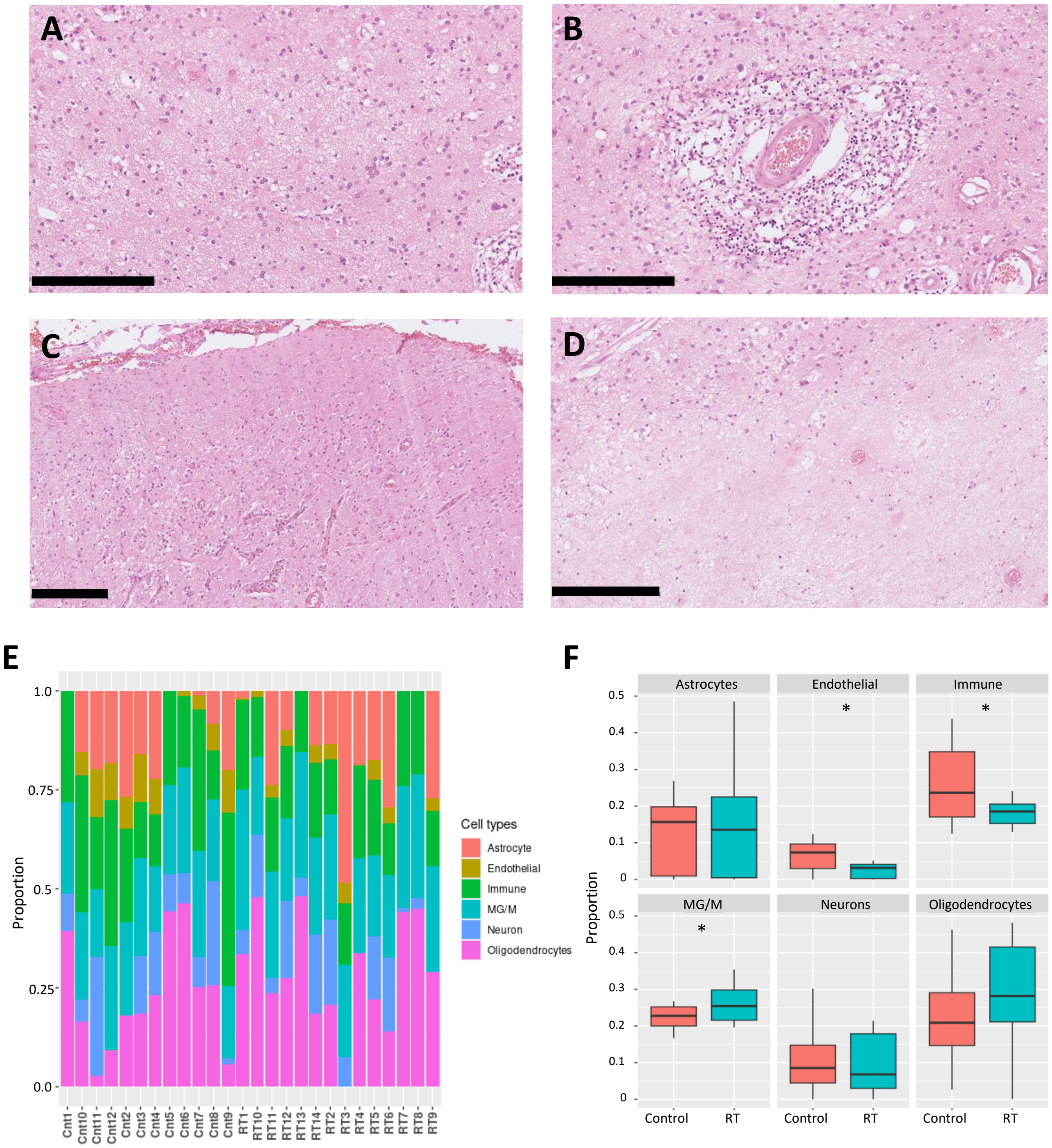

Supplementary figure 1: Representative histological images of irradiated brain from the study cohort are shown in (**A-D**): (**A**) shows reactive gliosis; (**B**) shows white matter inflammation and vasculopathy; (**C**) shows cortical vessel changes; (**D**) shows white matter rarefaction and necrosis. Scale bars represent 250 µm. (**E**) Cell-type deconvolution of DNA methylation data performed with methylCIBERSORT for each sample. (**F**) Box and whisker plots showing deconvolved cell-type proportions for control and RT samples, *P*-values were obtained using two sample t-test. **P* ≤ 0.05; ***P* ≤ 0.01

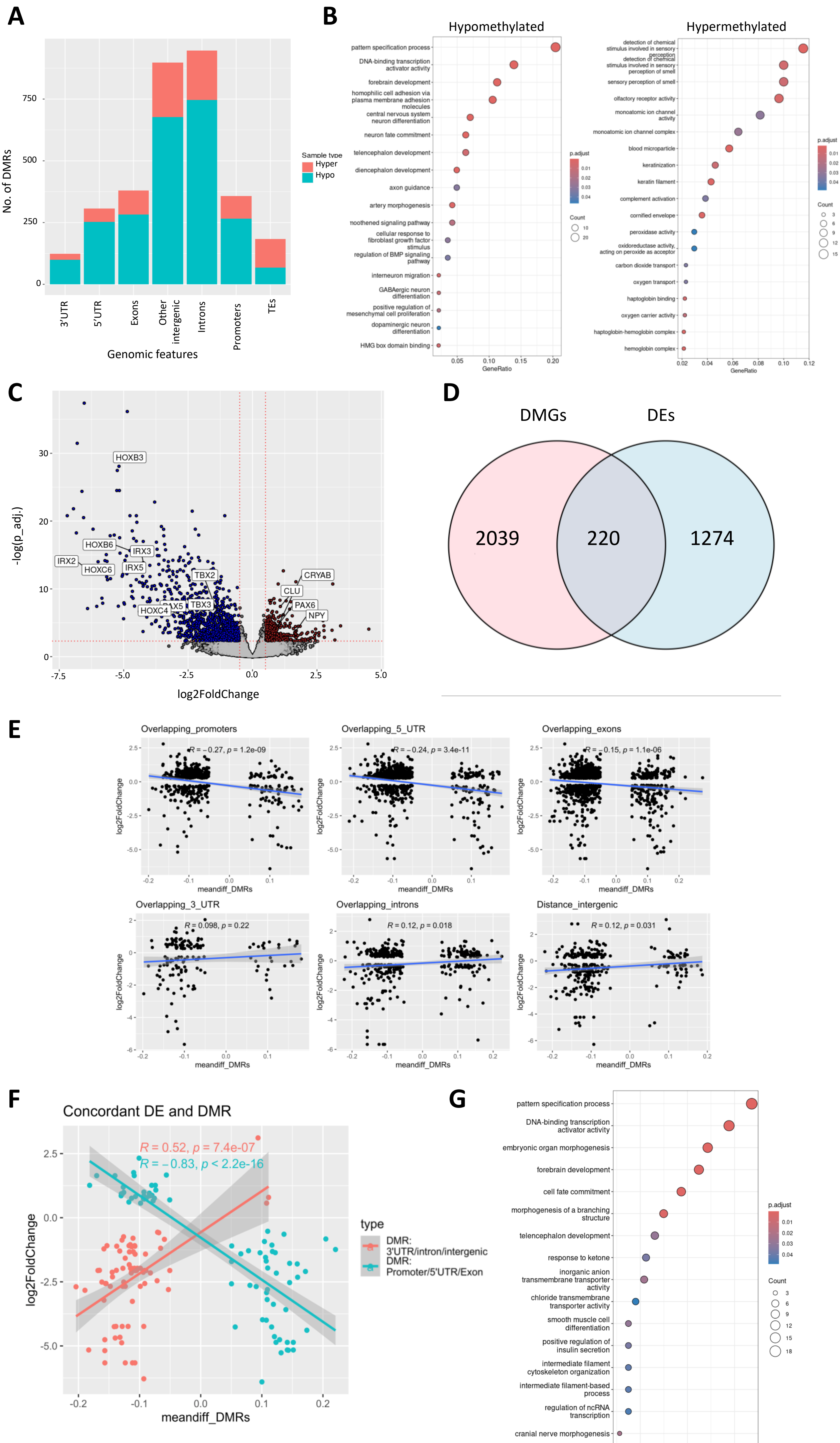

Supplementary figure 2

Supplementary figure 2: (**A**) Bar plot depicting the number of DMRs by genomic region, with proportion of hypermethylated (red) and hypomethylated (blue) DMRs; TE – transposable elements. (**B**) Selected GO pathways from top 150 DMGs hypomethylated at promoter/5'-UTR/exons and all GO pathways for top 150 DMGs hypermethylated at promoter/5'-UTR/exons. (**C**) Volcano plot of DE genes from RNAseq data between RT and control samples. Significant up-regulated genes in red and down-regulated in blue. Non-significant are marked in grey. Sig. cutoff of <0.05 p.adj < 0.05 and |log2FC| > 0.5. (**D**) Venn diagram of overlapping differentially methylated genes (DMGs) and differentially expressed genes (DEGs). (**E**) Scatter plots with regression line, showing differentially expressed genes by genomic annotation. (**F**) Scatter plot with regression of concordant genes identified from DE-DMR integration. (**G**) Selected pathways from GO pathway analysis of all concordant genes. *$P \leq 0.05$.

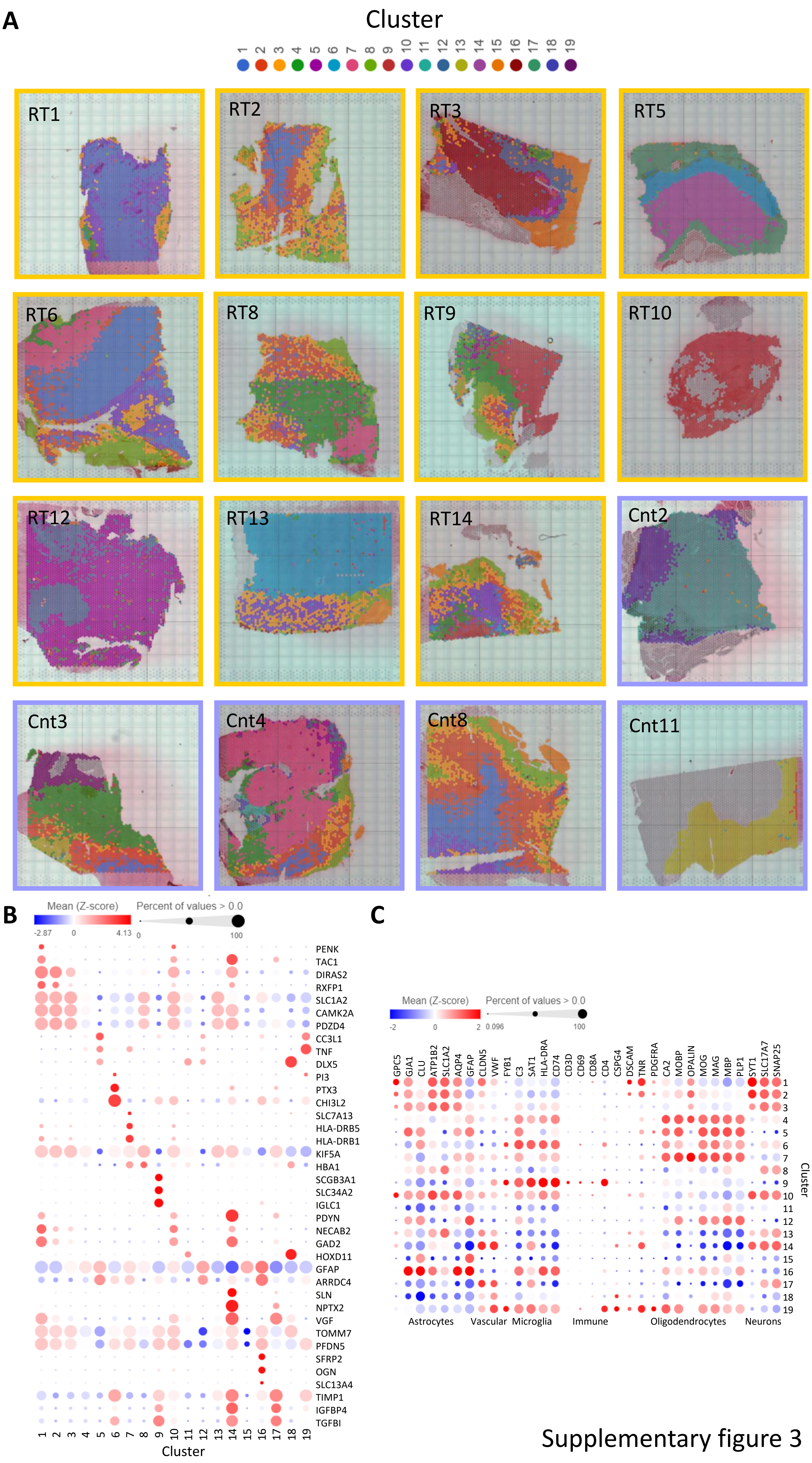

Supplementary figure 3

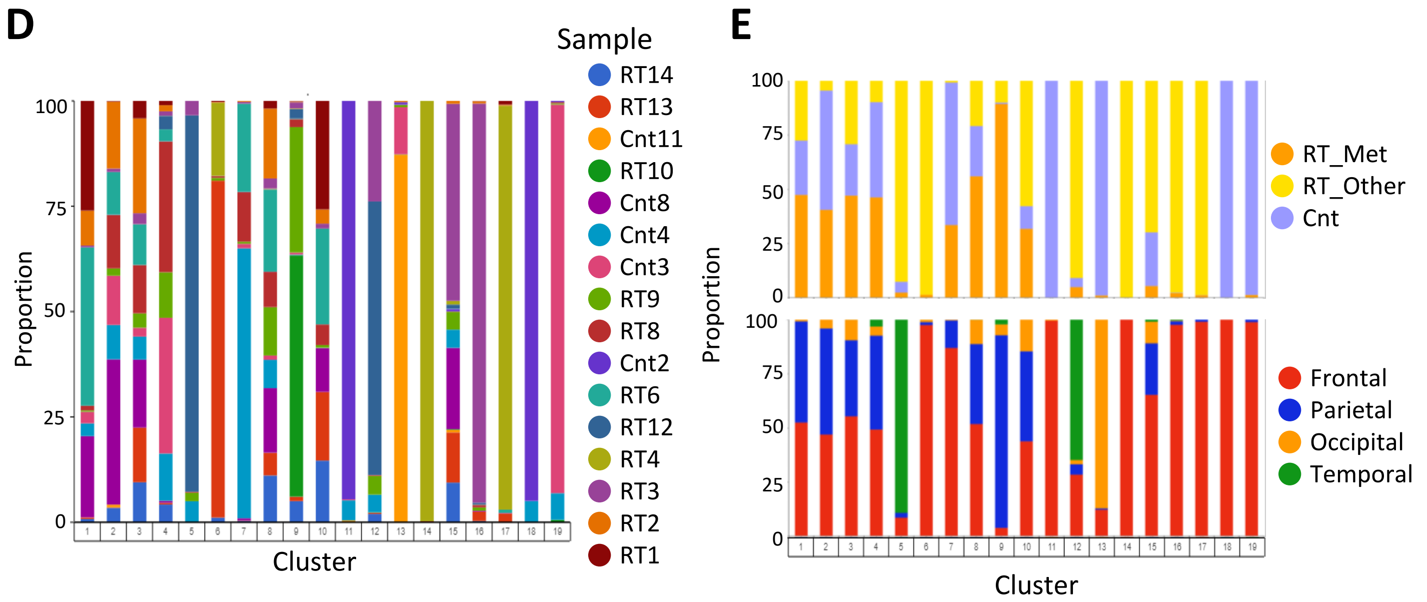

Supplementary figure 3 cont.

Supplementary figure 3: (**A**) Spatial transcriptomic slide overviews with spots overlying histology images. Spots are coloured by cluster, as in Fig. 3A. Border colour represents experimental group as in Fig. 1A. Dimensions of fiducial frame are 6.5 x 6.5 mm. Spots are no to scale. (**B**) Dot plot depicting selected differentially expressed genes for each cluster. Dot size corresponds to the percentage of spots expressing the gene in each cluster, and the colour represents the average expression level. (**C**) Dot plot depicting selected lineage marker genes for each cluster and associated cluster labelling. Dot size corresponds to the percentage of spots expressing the gene in each cluster, and the colour represents the average expression level. (**D**) Bar plot showing proportion of spots by sample for each cluster. (**E**) Bar plots showing proportion of spots by sample type (top) and brain location (bottom).

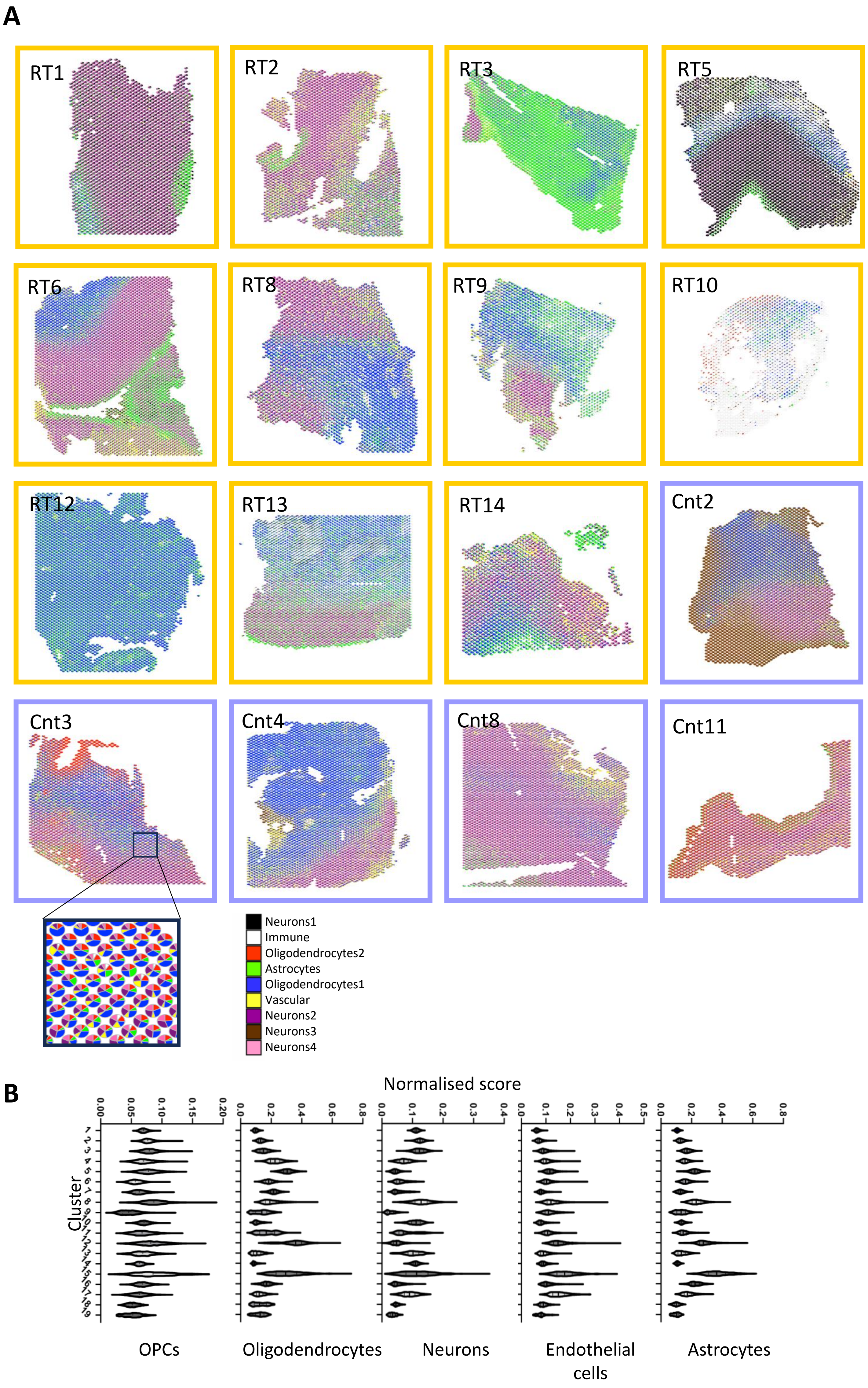

Supplementary figure 4: (**A**)Deconvolution of ST spots using STDeconvolve. Spot composition is visualised as a pie chart (shown enlarged in inset) and projected on the spatial coordinates. (**B**) Violin plots for cell type signature scores (from Al-Dalahmah *et al.*) for each cluster. Y axes show z-score normalised against gene expression per spot.

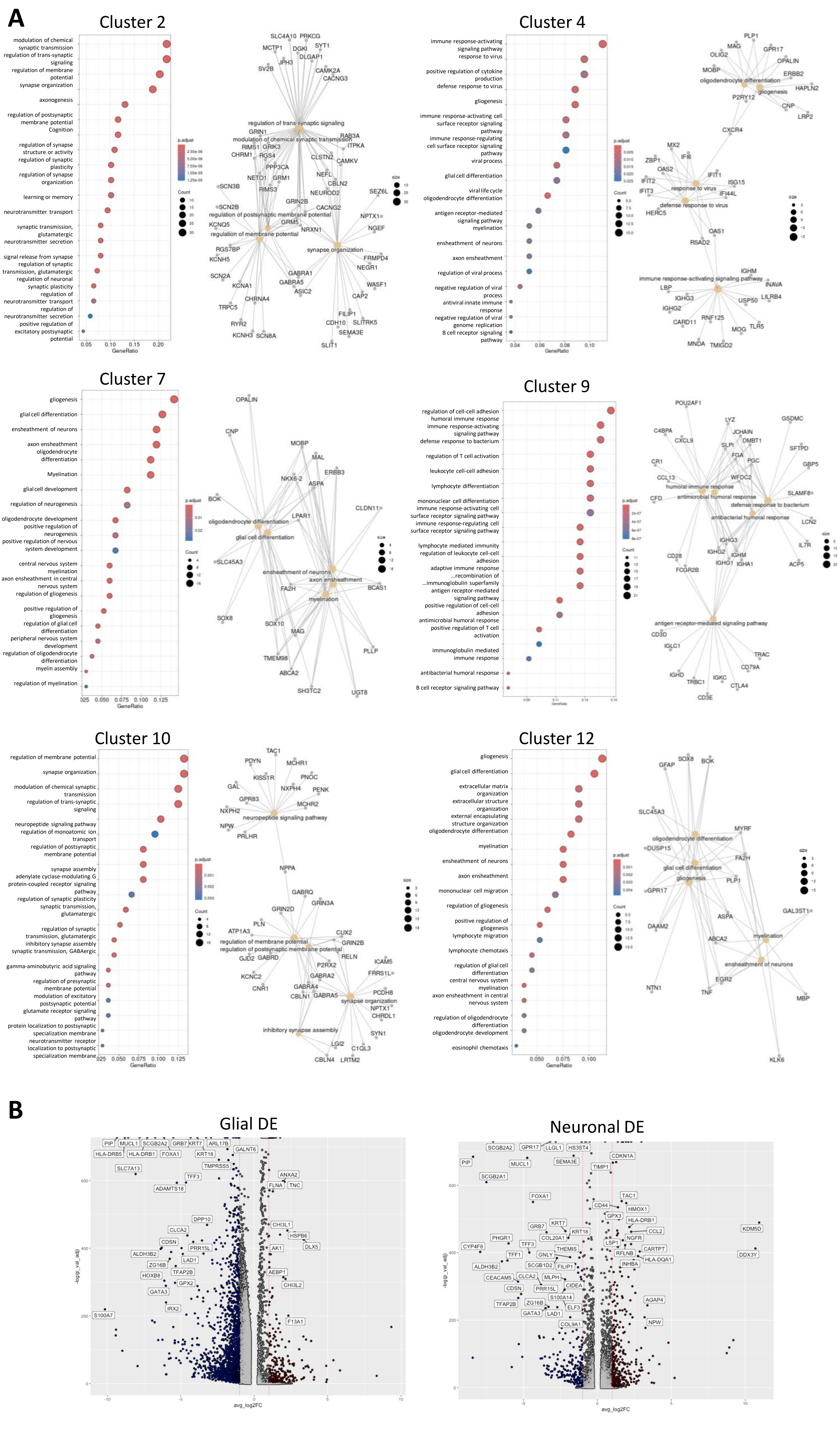

Supplementary Figure 5

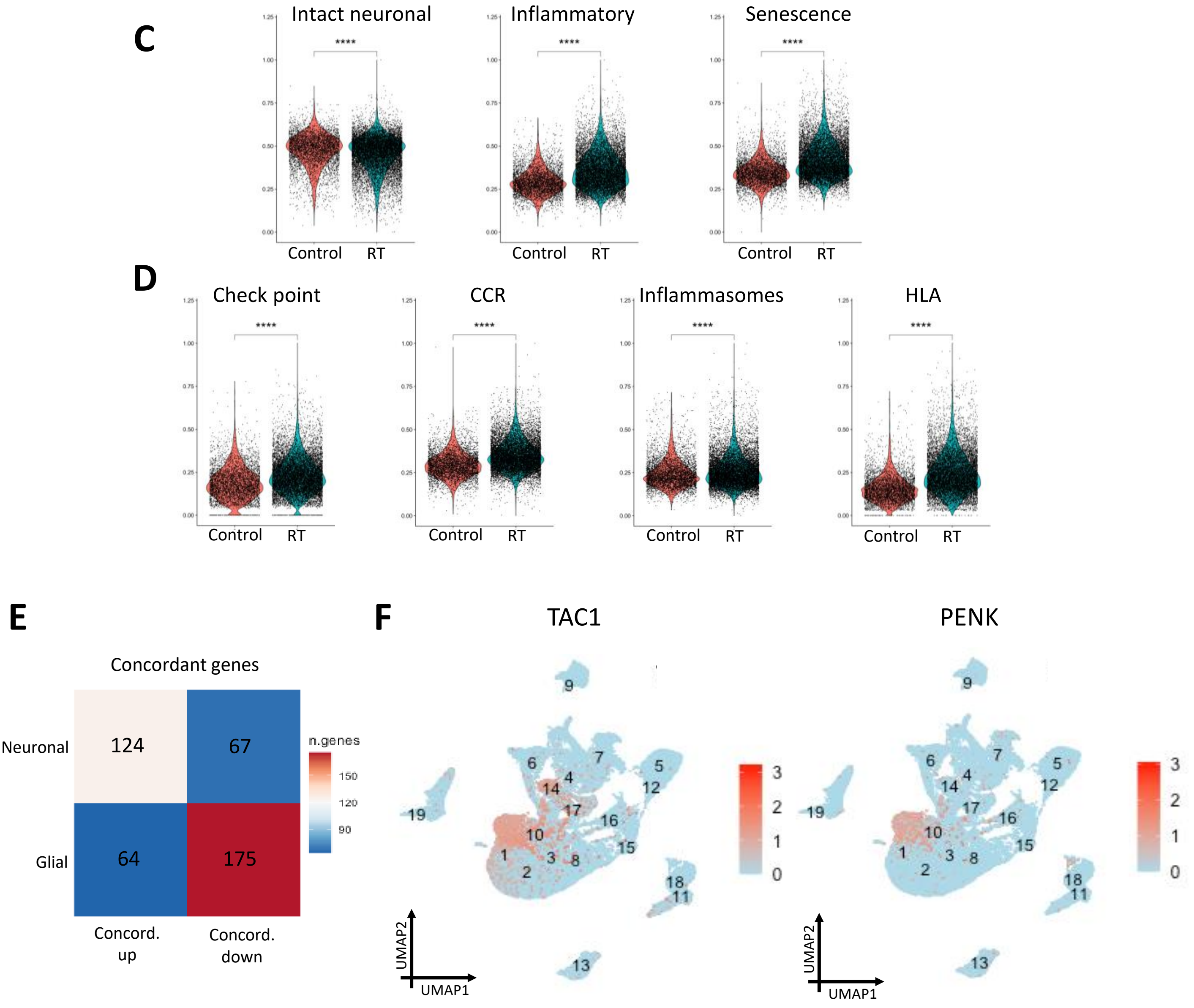

Supplementary figure 5 cont.

Supplementary Figure 5: (**A**) GO pathway analysis was performed for top 150 cluster markers and plots showing selected GO pathways for: neuronal clusters 2 and 10; glial clusters 4, 7; and 12 and immune active cluster 9 are shown. Size of dots and hubs corresponds to gene count for each pathway and edges represent single genes. See also supplementary data. (**B**) Volcano plots of DE genes between RT and control glial (left) and neuronal (right) ST clusters. Significant up-regulated genes in red and down-regulated in blue. Non-significant in grey. Sig. cutoff of <0.05 p.adj < 0.05 and |log2FC| > 0.5. (**C**) Violin plots for signature scores of all spots in neuronal clusters from control and irradiated samples for senescence, inflammation and intact neurons (see main text for details). Y axes show z-score normalised against gene expression per spot. Significance is tested with unpaired t-test. (**D**) Violin plots for signature scores of all spots in neuronal clusters from control and irradiated samples for components of the inflammatory pathway (see main text for details). Y axes show z-score normalised against gene expression per spot. Significance tested with unpaired t-test. Wilcoxon test used for (C) and (D); ****$P \leq 0.0001$. (**E**) Heatmap of numbers of genes with concordant expression/methylation from bullk DNAme and ST DEGs. (**F**) UMAPs of all ST spots showing PENK and TAC1 expression.

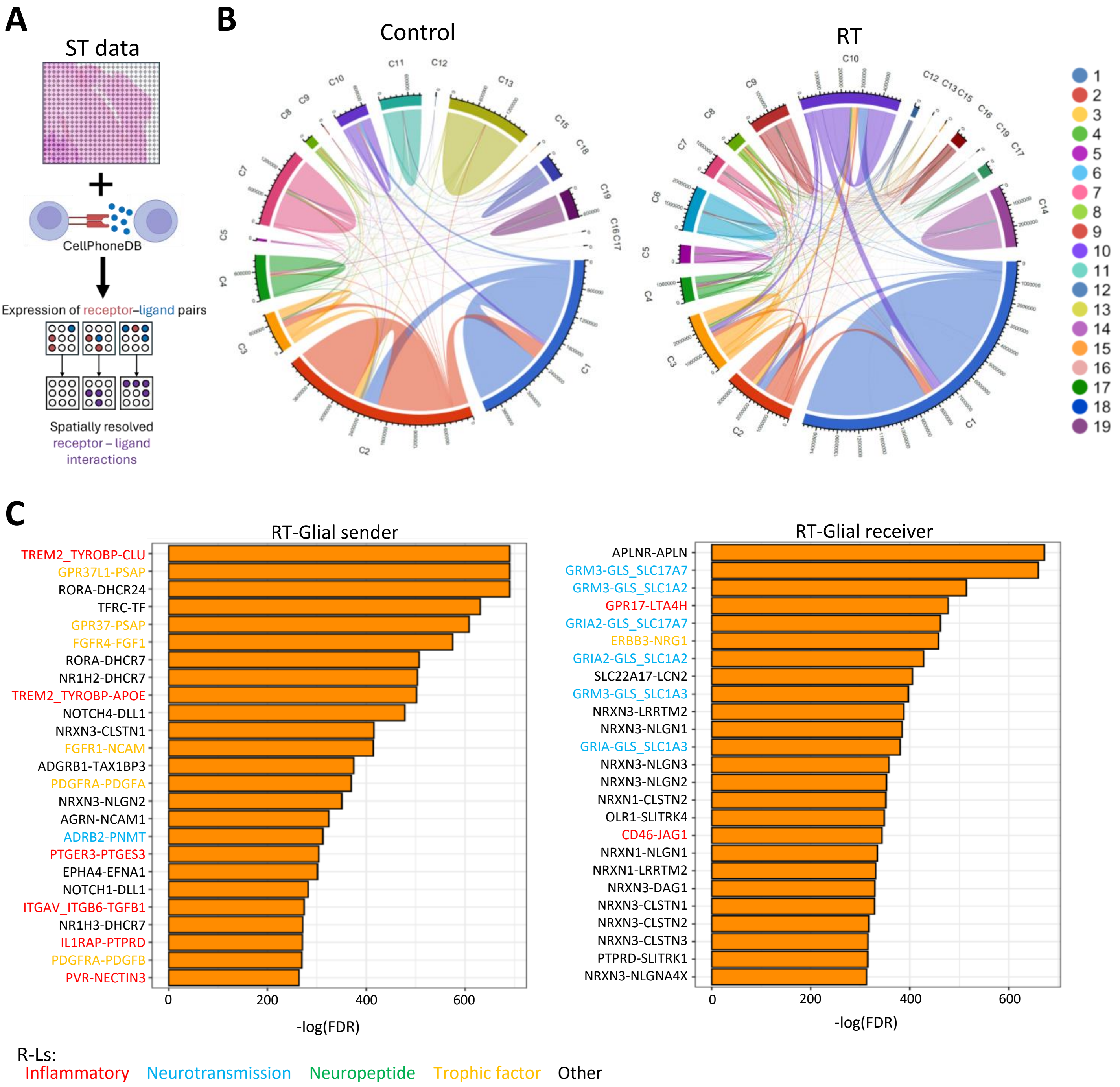

Supplementary Figure 6: (**A**) Schematic of generation of receptor-ligand (R-L) interaction scores. ST data was interrogated using a curated R-L database (CellPhoneDB[31]) to generate interaction scores where receptors and ligand were expressed in close proximity (also see Methods). (**B**) Circos plots showing interactions between all clusters using method shown in 5A. Chord thickness represents number of R-L interactions. (**C**) Bar plots showing 25 most significantly different R-L pairs when interaction scores were compared between control and RT glial clusters; see also supplementary data. Interaction scores between clusters were compared using a Wilcoxon Test and P-values adjusted for multiple hypothesis testing using the Benjamini-Hochberg procedure.

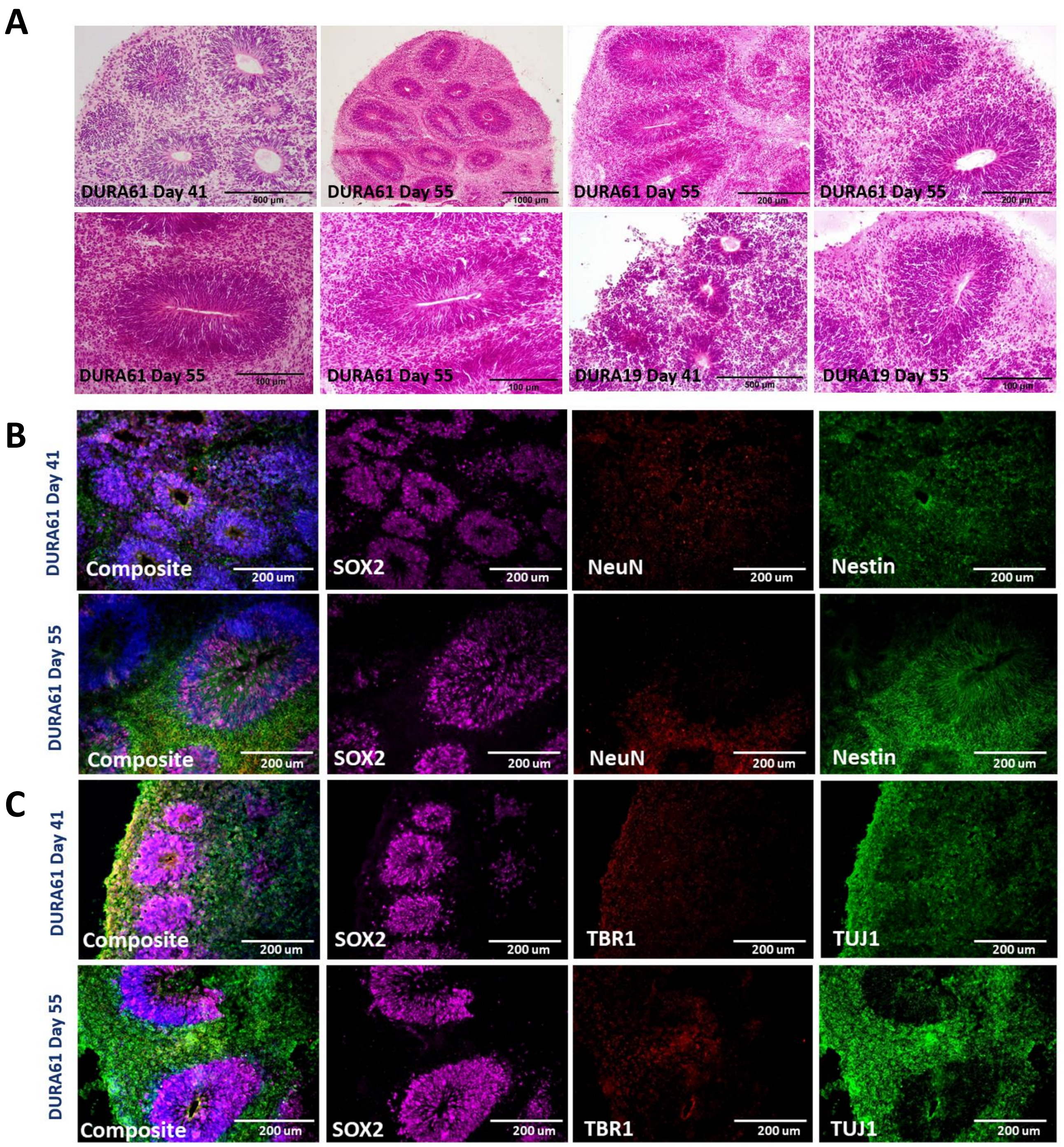

Supplementary figure 7: (**A**) H&E staining of COs from day 41 and day 55 of maturation from two patient-derive EPSC lines (DURA61 and DURA19). (**B**) Representative IF images of COs at day 41 and 55 stained with antibodies against SOX2, NeuN, Nestin and DAPI. Scale bars are 200 µm. (**C**) Representative IF images of COs at day 41 and 55 stained with antibodies against SOX2, TBR1, TUJ1 and DAPI. Scale bars are 200 µm

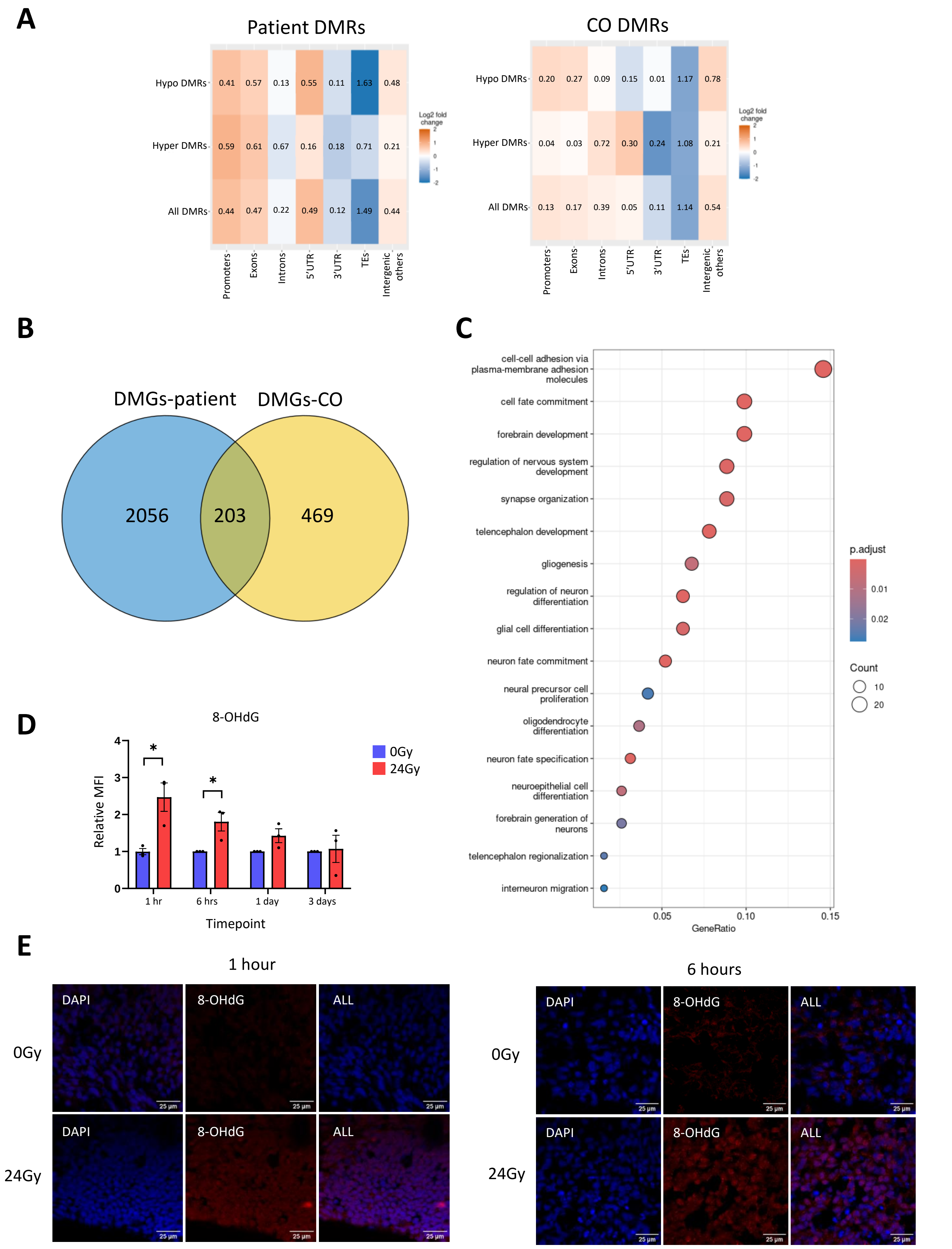

Supplementary figure 8: (**A**) Enrichment heatmaps for patient DMRs (left; see also Fig. 1E) and CO DMRs (right; see also Fig. 5D) showing genomic location of DMRs, for all DMRs, hyper- and hypomethylated DMRs, as compared to whole genome as represented on DNA methylation array. Scale represents log2FC(enrichment of genomic features in DMRs / whole genome). Numbers within boxes are Cohen's D effect size. Scale bars are 25 µm. (**B**) Venn diagram showing the overlap of DMGs from patient samples and CO model. (**C**) Selected GO pathways from DMGs shared between patient tissue and CO model. (**D**) Bar plot depicting relative mean fluorescence intensity (MFI) of 8-OHdG comparing irradiated organoids (red) to the control (blue) at timepoints ranging from 1 hour to 3 days post-irradiation. Each data point represents pooled data from a single CO. Unpaired t-test was performed to assess statistical significance. Error bars represent SEM. *$P \leq 0.05$. (**E**) representative IF images of 8-OHdG (red) at 1 hour (left) and 6 hours (right) post-irradiation. Nuclei were counterstained for DAPI (blue).

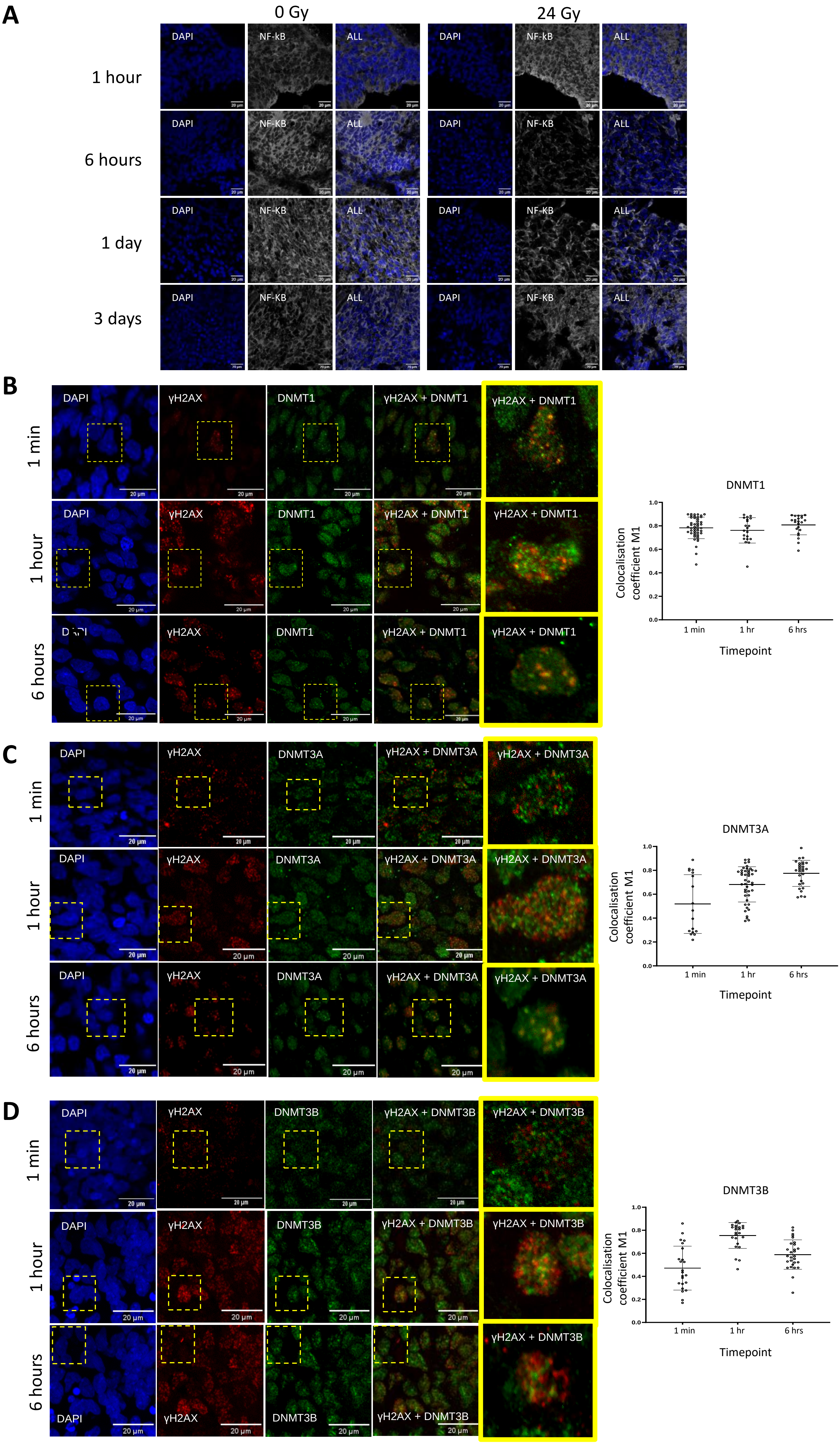

Supplementary figure 9

Supplementary figure 9: (**A**) Representative IF images of NF-kB p65 (white) at 1 hour, 6 hours, 1 day and 3 days post-irradiation. Nuclei were counterstained for DAPI (blue). Scale bas are 20 µm. (**B-D**) Left panels show representative immunofluorescence (IF) images illustrating colocalisation of γH2AX (red) with DNMT1 (**B**), DNMT3A (**C**) and DNMT3B (**D**) (all green) at 1 minute, 1 hour and 6 hours post-irradiation. The overlay images demonstrate points of colocalisation in yellow. Nuclei were counterstained for DAPI (blue). Scale bars are 20um. Right panels show dot plots of Manders colocalisation coefficient (M1) value from individual nuclei displayed from organoids at each timepoint. The error bars represent SD.

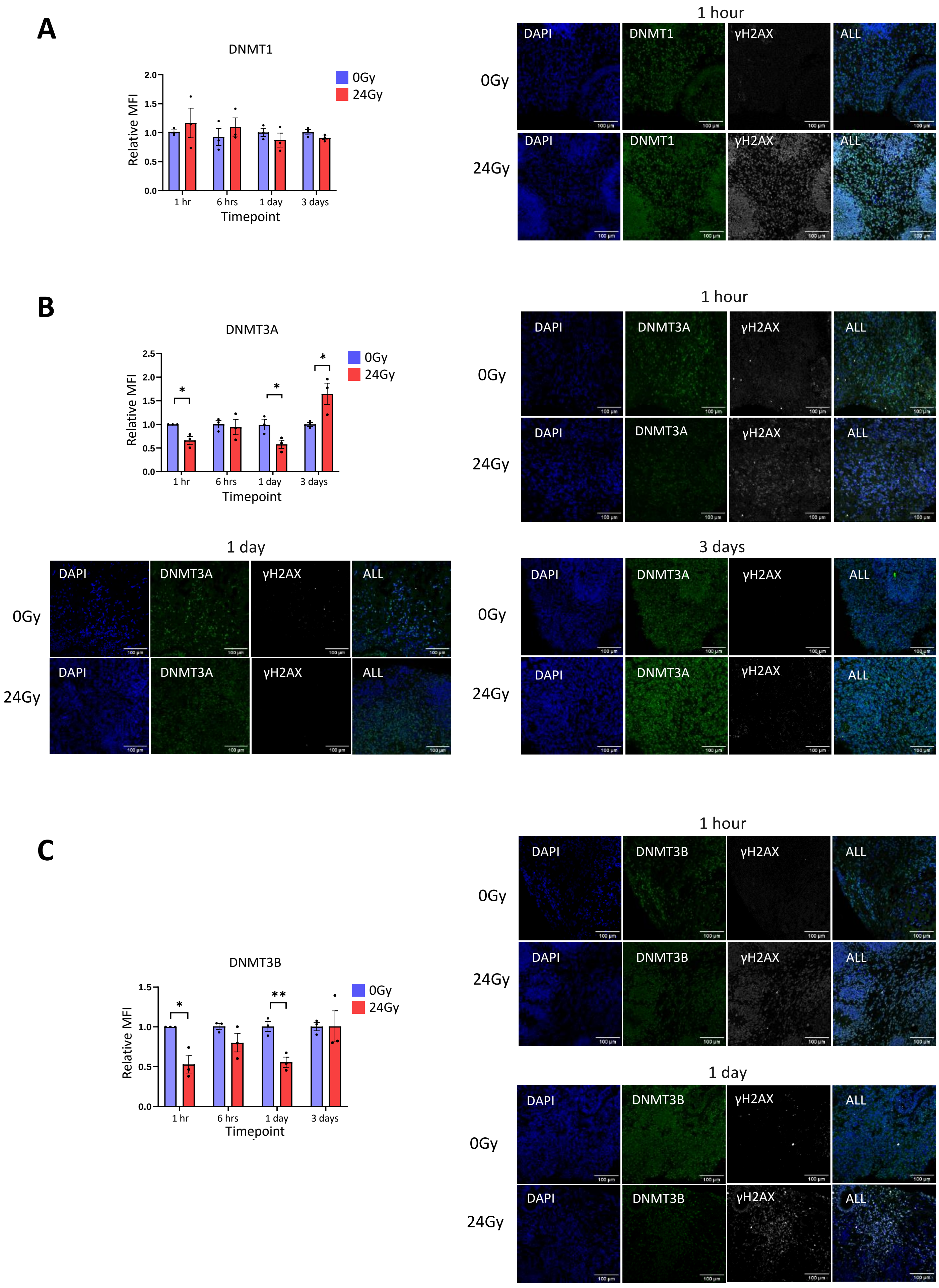

Supplementary figure 10: Top left panels are those seen in figure 6G, showing bar plots depicting relative mean fluorescence in tensity (MFI) of DMNT1 (**A**), DNMT3A (**B**) and DNMT3B (**C**) at timepoints ranging from 1 hour to 3 days post-irradiation. Each data point on the graph represents pooled data from a single CO. Unpaired t-test was performed to assess statistical significance. Error bars represent the standard error of the mean (SEM). Other panels depict representative IF images of DMNT1 (**A**), DNMT3A (**B**) and DNMT3B (**C**) (all green) and γH2AX (white) at various timepoints post-irradiation. *$P \leq 0.05$, **$P \leq 0.01$.

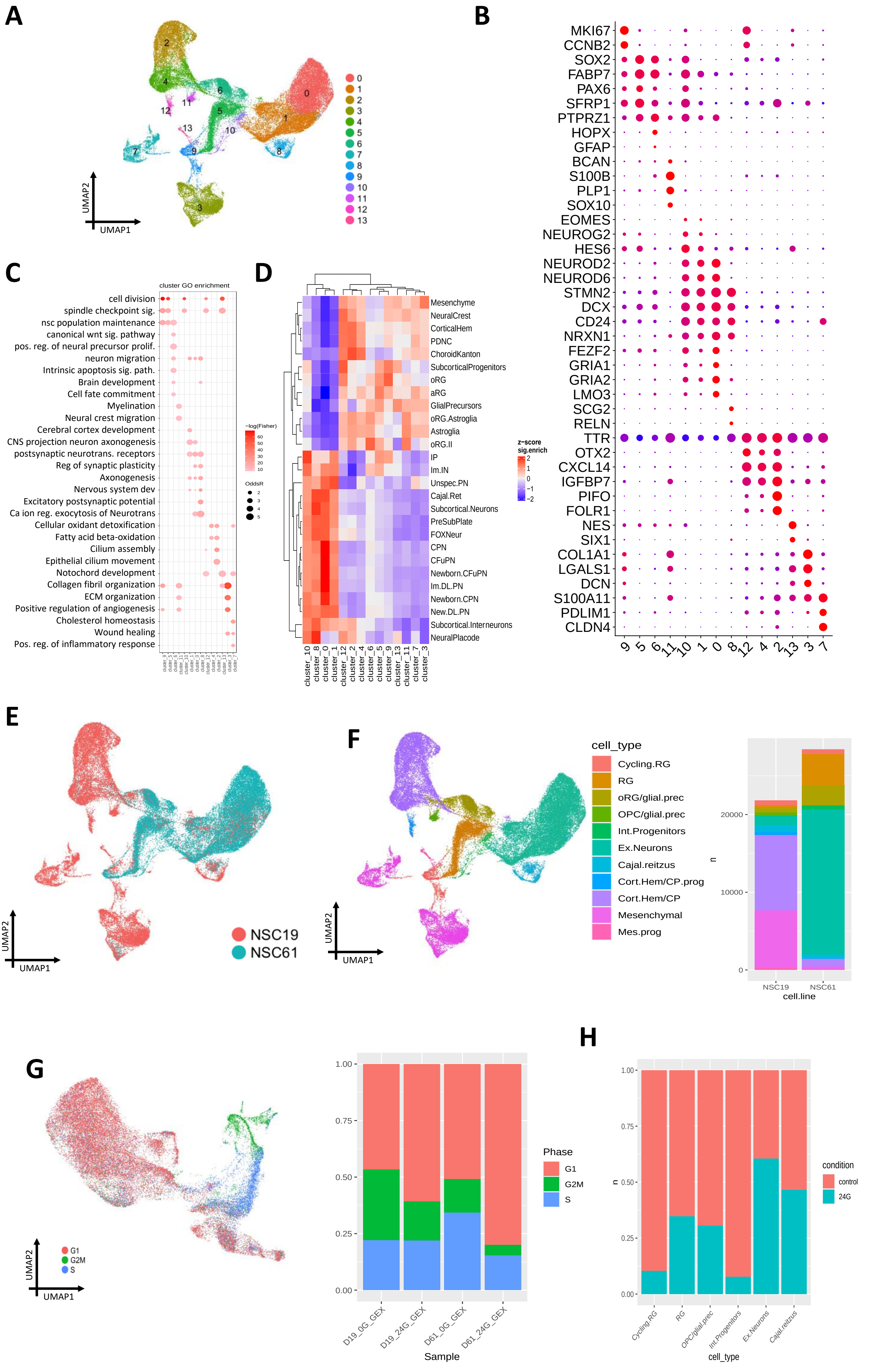

Supplementary figure 11

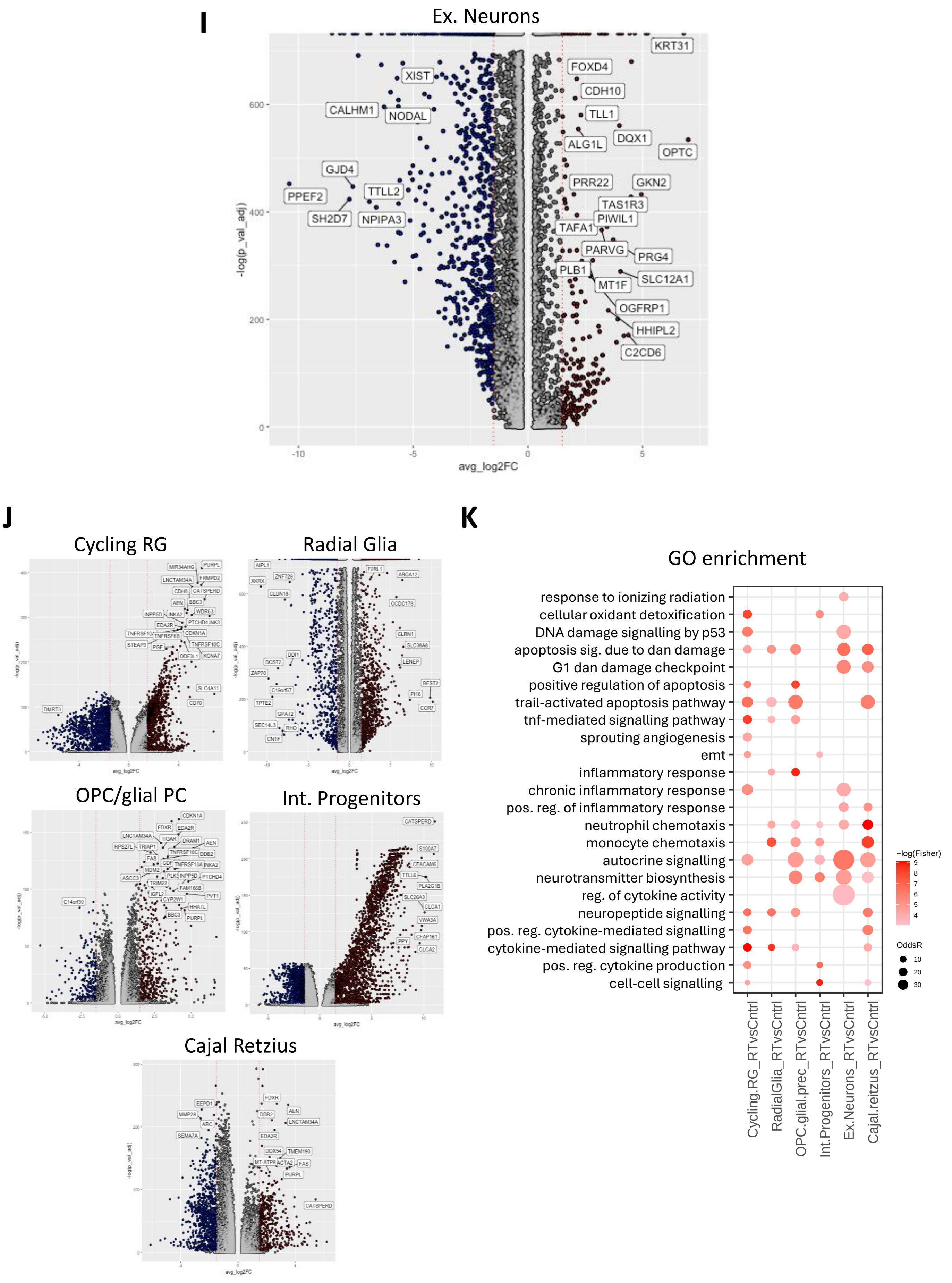

Supplementary figure 11 cont.

Supplementary figure 11: (**A**) UMAP of all CO single cells coloured by cluster. (**B**) Dot plots of cluster marker gene expression used for cell type classification. (**C**) Dot plots of cluster GO enrichment. (**D**) Heatmap showing z-scores of signature enrichment for cell types from Uzquiano et al. (**E**) UMAP of all CO single cells coloured by patient-derived NSC origin. (**F**) UMAP of all CO single cells coloured by annotated cell type (left) and bar plot showing cel type by patient-derived NSC origin. (**G**) UMAP of neuroglial cells coloured by cell cycle phase (left) and bar plot showing cell cycle phase by sample (right). (**H**) Bar plot showing proportion of neuroglial cells from control and 24 Gy. (**I**) Volcano plot of DE genes for irradiated and non-irradiated ex. neurons. Significant overexpressed genes in red and down-regulated in blue. Non-significant are marked in grey. Sig. cutoff of p.adj < 0.05 and |log2FC| > 1.5. (**J**) Volcano plots of DE genes by other neuroglial cell types with significance cutoff of <0.05 p.adj < 0.05 and |log2FC| > 1.5. (K) GO enrichment for all neuroglial cell-types.
