# Supplementary material for: Disruption of DNA methylation underpins the neuroinflammation induced by targeted CNS radiotherapy
Source: Brain. 2025 Apr 29;148(9):3137–52. doi: 10.1093/brain/awaf163 (PMC12404709; doi:10.1093/brain/awaf163)
Supplement: awaf163_Supplementary_Data [file awaf163_supplementary_data.zip › brain-2024-01691-File014.pdf]

Materials and methods

Study cohort

Tissue samples were identified by medical records searches and slide review by a neuropathologist. We retrospectively identified formalin fixed paraffin embedded (FFPE) neurosurgical samples from patients that had undergone supratentorial targeted radiotherapy, followed by resection and that contained peri-lesional brain tissue within 15 mm of the irradiated lesion, that would have been irradiated as part of the treatment field in our centres, as confirmed by clinical oncologist. Characteristics of all patients included in the final analysis, whose samples yielded suitable quality and quantity of DNA and RNA, are summarised in table 1. Ethical approval was secured for the use of all patient samples (BRAIN UK Ref: 20/005).

DNA and RNA extraction from FFPE tissue

H&E and subsequent unstained sections were attained from the representative FFPE blocks. Concentrations and quality of DNA and RNA were quantified using the Nanodrop 1000. DNA methylation array was performed by UCL Genomics, UK and RNA transcriptome analysis by Oxford Genomics, UK. For DNA methylation, oxidative bisulfite chemistry and bisulfite conversion was applied alongside Infinium Methylation EPIC (850K) BeadChip Array for the detection of 5-methylcytosine (5mC). Transcriptome analysis involved conversion of selected mRNA to cDNA. The prepared libraries are size selected and multiplexed before 150bp paired end sequencing on the Illumina platform (NovaSeq6000). A minimum concentration of 500 ng for genomic DNA in 45 µl and 200 ng for RNA in 12 µl with 260/280 and 260/230 ratios over 1.80 was used. Samples that failed to meet these criteria were selected for further concentration and purification.

DNA methylation analysis

DNA methylation was assessed with Illumina Infinium Methylation EPIC arrays. Beta values were extracted with the R package wateRmelon[1]. Probes associated with the sex, mitochondrial chromosome (i.e., chrX, chrY and chrMT), single nucleotide polymorphism (SNPs) and the probes that fail in at least half of the samples (i.e., p-value > 0.05 in more than 13 FFPE samples, more than 4 CbO samples) were filtered out. Additionally, adjusted Dasen

normalisation was performed on the beta values to further reduce bias. All analyses were carried out using the reference genome hg38.

**Cell types**	**Source**	**Number of samples**
B-cell, CD4T, CD8T, NK, Eosinophils	Jaffe 2024[2]; Gervin et al. 2024[3]	74
Neurons, oligodendrocytes, astrocytes, microglia	Hannon et al. 2024[4]	76
Regulatory T-cells (Treg)	Zhang et al. 2013[5]	4
Endothelial	Cvitic et al. 2018[6]	30
Microglia (additional)	De Witte et al. 2022[7]	56

Supplementary table 1: Details of cell types, sources and sample numbers used for reference-based deconvolution of patient DNA methylation data.

Reference-based deconvolution of DNA methylation data

Reference probe sets for known brain cell types were generated from 240 samples from published DNA methylation data (Supplementary table 1 The common probes among these 240 samples were selected, and the basis probes (n=2146) for the cell-type deconvolution were selected using the R function feature.select.new[8], which is a support function similar to the feature.select function within the R package methylCIBERSORT Finally, the deconvolution task was carried out with CIBERSORTx[9] and the proportions of different cell types in different samples were obtained (Supplementary Fig. 1E-F).

DMP, DMR and DMG calling

Differential analysis was performed to obtain DMPs and DMRs by comparing the irradiated samples with the control. The statistical model used for DMP calling was the limma linear model, the built-in method within the cpg.annotate() function from the package DMRcate. Age, gender, sample type, deconvolved cell type proportions, and time post-RT in months were used as covariates in the design matrix for DMP calling in patient samples, and sample type only for CO samples, with the FDR value set to 0.01 for identification. DMPs where then used to call DMRs.

To qualify as a DMR, the criteria were that the DMRs should contain at least 3 CpG sites and be separated by no more than 400 base pairs, and a cutoff of |meandiff| > 0.05 was used. Meandiff of a DMR was calculated such that, once a region (i.e., DMR) is demarcated, the probes it covers are identified, the mean beta values of these probes in Control and experimental samples were then calculated, and the meandiff would be the difference between these two mean beta values. Annotation of DMRs to genomic locations was performed using the GenomicRanges R package and the annotation file Homo_sapiens.GRCh38.cdna.all.fa.gz, release 110, downloaded from Ensembl. The coordinates of different genes and their genomic features were first extracted from the annotation, then the DMRs were mapped to locations within genes, or we consider the DMR as intergenic and assign it to a gene within 50 Kb of the TSS.

DNA methylation exploratory analyses

Hierarchical clustering was performed using the R package ComplexHeatmap and 2D/3D principal component analysis (PCA) with the R package ggbiplot to perform exploratory analysis. Further analysis was performed to determine the genome regions affected: the significance of each genomic feature's enrichment or depletion within the different types of DMRs compared to the whole genome captured by the EPIC array was calculated using Cohen's D, which is a method frequently used to determine to effect size. In our analysis, genomic features with their absolute Cohen's D value greater than 0.4 were considered as showing significant enrichment or depletions in FFPE samples. Differentially methylated genes were therefore acquired for subsequent analysis.

RNA sequencing analysis

The raw RNA-seq data was examined using FastQC v0.12.14 and thereafter trimmed using TrimGalore v0.6.105. Alignment to the reference genome (Homo_sapiens.GRCh38.cdna.all.fa.gz, release 110) was performed using Salmon v1.10.2. R package trimeta 7 was utilised to read and normalise the raw counts, followed by conversion of the normalised data from the transcript level to gene level. Genes with no counts or only one count across samples were filtered out. Normalised raw counts were further converted to counts per million (CPM) and genes reporting 0 CPM in more than half of samples were filtered out. Differential expression analysis was carried out using the package DESeq2, and significant differential expression was considered with $p.adj < 0.05$ and $|log2FC| > 0.5$.

Integration and concordant genes

Correlation between differentially expressed genes and DNA methylation in individual genomic locations in patient data showed a significant negative correlation between expression and promoters, 5'UTRs and exons, and a positive correlation between expression and 3'UTRs, introns and all intergenic regions, which was significant for introns and intergenic regions (Supplementary Fig. 2E). Therefore, concordant genes were defined as those genes that have anti-correlated expression and promoter/5'UTR/exon methylation, and correlated expression and 3'UTR/intron/intergenic methylation.

Pathway analysis and GSEA

Unless otherwise stated, Gene Ontology (GO) pathway analysis was performed using the R package clusterProfiler[10] and GSEA analysis was performed using the R package fGSEA v4.4 (Korotkevich et al. 2019) to identify the enriched GO gene sets. See figure legends for details. For analysis of concordant genes, to avoid calling pathways on opposingly ranked lists only DMGs with promoter/5'-UTR/exonic methylation were used, since they have the much stronger relationship to expression.

Spatial transcriptomics

11 RT cases and five control samples were used for analysis. The criteria used for selection of cases for spatial transcriptomics was a combination of measurement of RNA quality by DV200

in combination with neuropathological assessment to locate areas that had cortex and WM with preserved spatial continuity to fit within the fiducial frame of the Visium slide (6.5x6.5mm^2). Each region was sampled with up to 5000 spatially barcoded RNA-capturing spots with the Visium platform. The cases with the best preservation of brain architecture were chosen for spatial transciptomic analysis. The mean number of spots under tissue was 2701, giving a total of >40,000 spots, with a mean of 4436 unique molecular identifiers recovered and 17,608 genes detected per sample. After removal of spots containing tumour cells, by neuropathological assessment and computational filtering, 39,151 spots were included in the final analysis. Case numbers used and numbers of spots assessed were comparable to published literature[11-14]. Spatial transcriptomics (ST) of 6.5x6.5mm^2 areas was conducted using 10X™ Visium Spatial Gene Expression Slide & Reagent Kit, 16 rxns (PN-1000184), according to the protocol detailed in document CG000239 available in 10x demonstrated protocols. 10 micron-thick tissue sections were mounted on the ST slides and stained with H&E described in document CG000160 available in 10X demonstrated protocols. Imaging of whole slides was done at 20X magnification on a Nikon Eclipse 80i Stereology Microscope. The remaining steps were conducted according to the manufacturer's protocol. The libraries were sequenced on multiple Illumina Nextseq 2000 (paired end dual-indexed sequencing) flowcells to achieve the recommended number reads per ST spot. The ST samples were prepared using 10X genomics Space Ranger software, which uses tiff image files of tissue, raw fastq sequencing files and 10X Visium slide information to align raw reads to the reference genome and output count matrices and annotated tissue section images. The reference genome used for alignment was built from Ensemble GRCh38 version 91 assembly. All other parameters for generating the counts data for ST were set to default settings. Slides with overlying spots were visualised in Partek Flow (Illumina). CPM normalisation was performed and after removal of any spots containing tumour cells, by neuropathological assessment and computational filtering for tumour cells that had not been removed manually. PCA was performed for dimensionality reduction, and output used for unsupervised clustering using the SLM method with default settings other than a resolution of 0.75 and PC of 5. Unsupervised clustering was then visualised as a UMAP using default Seurat v5[15] settings with 10 principle components. Bubble plots were generated in Partek Flow using the hierarchical clustering / heatmap function, using normalised count matrices. Differential expression analysis between clusters was performed using the GSA algorithm in Partek Flow using default settings. Spatial plots with overlying clustered spots in supplementary figure 3A were generated in Partek Flow using the Visium plot function. To perform differential expression between neuronal (clusters 8,3,10,2,1 & 14)

or glial (clusters 15,16,12,5,7 & 4) niches, cells were separated on the basis of experimental condition and differential expression was calculated using a Wilcoxon Rank Sum test. Gene set enrichment analyses on differentially regulated genes was then performed using the fGSEA v4.4 R package[16], and gene sets were accessed from MsigDB[17].

Signature scores

Signatures were extracted from the cited literature uploaded to Partek Flow using the List function. Scores for each spot were calculated in Partek Flow. Scores were then normalised for gene count per spot and truncated violin plots produced using GraphPad Prism and significance calculated with unpaired t-test.

Reference-free deconvolution

Spatial deconvolution of tissue spots was performed using the STDeconvolve package[18]. In brief, h5 files and spatial images and coordinates produced by the 10X Space Ranger pipeline were used as inputs. Samples spots were filtered as above and count matrices and tissue spot positions were then extracted from each sample. Further filtering was performed to remove poor barcodes and features using the STDeconvolce cleanCount function, the following parameters were used: min.lib.size = 250, min.reads = 10, min.detected = 5. Next the top 500 over-dispersed genes in each dataset were identified and then the common over-dispersed genes shared by all samples were identified. All sample count matrices were then merged into a single count matrix based on common over-dispersed genes. LDA models with k varying between 2 and 18 were fitted to the merged count matrix. The optimal k was found to be 9 and results were plotted using STDeconvolve functions. Markers of each potential cell type were identified to determine the identities and finally transcriptional profiles of each of the potential cell types compared. Cell types were annotated using a combination of the most highly expressed genes, using the Human Protein Atlas and STAB database[19], and the spatial location, confirmed by a neuropathologist.

Correlation heatmap

To group similar clusters, we computed cluster gene specificity scores for all cluster marker genes (mean normalised counts per cluster/total mean normalized counts) – and performed Pearson correlation between cluster gene specificity signatures, representing data as a heatmap.

Receptor-ligand interactions

We derived interaction scores based on the mean expression values of R-L pairs expressed in close proximity to each other and took into account the expression of co-factors and multi-unit receptors for best functionality results. Custom code was written to compare the interaction scores of receptor-ligand pairs between clusters, based on the work from Kaufmann et al.[11]. In brief, the expression of each receptor and ligand, listed in the CellPhoneDB v5[20,21] database, in each tissue spot was determined. In the case of receptor complexes the expression value of the least expressed subunit was taken as the expression value. If any subunit was not expressed the expression value of the complex was set as 0. Next interaction scores were determined for each tissue spot, and its immediate surrounding neighbours. Interaction scores were also calculated within a tissue spot. The interaction score was defined as the mean expression of receptor A and ligand B in tissue spot 1 and tissue spot 2 respectively. Receptor expression was always taken from spot 1 – therefore spot 1 was the receiver of signalling, whilst ligand expression was always taken from spot 2; therefore spot 2 was the sender of signalling. This calculation processes was iterated across all tissue spots in combination with all neighbours of each tissue spot. Interactions were labelled using the following nomenclature: signalling_pathway_name-receptor_complex-ligand. In cases where receptor-ligands are not associated with a signalling pathway the naming nomclature is: -receptor_complex-ligand. Once interaction scores were calculated each tissue spot was annotated with its cluster identity. In this way, the interaction scores for a given cluster could be extracted. Extracting interaction scores based on the spot 1 cluster annotation identified the interaction scores that spots for a given cluster were receiving, whilst using the spot 2 cluster annotation identified the interaction scores that spots for a given cluster were sending, because receptor expression was always taken from spot 1 whilst ligand expression was always taken from spot 2. Finally, interaction scores for each receptor-ligand pair detected were compared between two clusters, in terms of both what each cluster was sending and receiving. Interaction scores were compared between irradiated and non-irradiated neurons and glia, as described in the main text, using a Wilcoxon Test and p-values adjusted for multiple hypothesis testing using the Benjamini-Hochberg procedure. All code produced for this spatial receptor-ligand neighbourhood analysis has been deposited on Github (https://github.com/jamesboot/interactVis).

Cerebral organoid culture

Cerebral organoids (COs) were established from EPSCs derived from fibroblasts of dura mater from two different patient lines: DURA19 and DURA61[22]. When the cells reached 70% confluence, they were harvested for the development of COs. The number of viable cells was calculated using a haemocytometer and trypan blue staining. 70,000-80,000 EPSCs were used for the development of individual organoids using the STEMdiff™ Cerebral Organoid Kit (Stemcell technologies, #08570) and the STEMdiff™ Cerebral Organoid Maturation Kit (Stemcell technologies, #08571), following the producer's protocol. Briefly, the cells were transferred into embryoid body (EB) media to aid the development of embryoid bodies in round bottom ultra-low attachment 96 well plates (Costar, #7007). During the fifth day of the development process, when the EB diameter had grown to a minimum of 300µm, the EB bodies were cultured in 24-well ultralow attachment plates (Corning®, #3473). STEMdiff™ Cerebral Organoid Basal Medium 1 and Supplement B were used to initiate the development of the neural ectoderm layer. ROCKi was also incorporated in the media during the first four days of culture. Once neuro ectoderm development was successful, the EBs were embedded in Matrigel droplets on day 7 and cultured in neural expansion media to encourage the neuroepithelial bud expansion. Parafilm (Sigma-Aldrich, # HS234526B) was sterilised and used as embedding surface. Circular indentations were created on a sterilised parafilm contained in a 10mm dish. Each EB was placed onto individual indentations and any surplus media was removed before adding Matrigel (Corning®, #354277). The EBs are immersed in Matrigel and incubated at 37ºC to allow the gel to set. Next, the structures were carefully washed off from the parafilm with neural expansion media (STEMdiff™ Cerebral Organoid Basal Medium 2, Supplement C and Supplement D) and transferred onto 6-well ultralow attachment plates (Corning®, #3471), with approximately ten EBs per well. Finally, the organoids were cultured in CO maturation media (STEMdiff™ Cerebral Organoid Basal Medium 2 and Supplement E) on day 10 and transferred to an orbital shaker at 100 RPM for the remaining culture period to further aid neural tissue growth and development. The organoids underwent media change every 3 to 4 days in the maturation media and reached maturity on day 30. Irradiation was performed using X-Ray Biological Irradiator within the Queen Mary Biological Science Unit and radiation dose of 24 Gy was administered at 8.5Gy/min.

Single-cell RNA sequencing

For scRNAseq experiments, CO from both the two patient derived lines described above were used, and for control (0 Gy) and irradiated (24 Gy) conditions, 3-5 COs for each condition were collected at 24 hours after irradiation and pooled for processing. COs were then processed with the Brain Tumour Dissociation Kit (P) (Miltenyi Biotec,130-095-942) following the manufacturer's protocol and using the gentle MACS dissociator. 3-5 pooled COs derived from gene-edited EPSC were harvested and washed with D-PBS without calcium and magnesium. The organoids were then transferred into a C-Tube containing the pre-heated enzyme mix 1 followed by the gentleMACS Program m_brain_01, 37ºC incubations under slow continuous rotation, gentleMACS Program m_brain_02, addition of enzyme mix 2 and gentle MACS Program m_brain_03. Single-cell suspension was passed through a 70µm strainer and centrifuged at 300g x for 10 minutes. CO cells were then resuspended in 0.04% BSA in DPBS, and viability and cell number were assessed with the Countess® II FL Automated Cell Counter using trypan blue. 100,000 cells were aliquoted into a new tube in 100µl to achieve a cell density of 1000 cells/µl as required by the 10X Genomics Single-cell RNA sequencing protocol, followed by a final count to ensure the correct number of cells.

Library preparation was performed at the Single Cell Genomics Facility (UCL, Cancer Institute) using the Chromium Next GEM Single Cell 3' Kit v3.1 (10X Genomics, 1000268) along with the Chromium Next GEM Chip G Single Cell Kit (10X Genomics, 1000120) according to the manufacturer's instructions. Pooled samples libraries were sequenced with lane sequencing on Novaseq 6000 S4 (Novogene). The Cell Ranger 2.0.1 pipeline was used to align reads to the GRCh38 human reference genome and produce count matrices for downstream preprocessing and analysis using the Seurat v5.0 R package[13]. nCount_RNA < c & nFeature_RNA > 500 & nFeature_RNA < 6500 & percent.mt < 15.

For quality filtration, cells with 500-6500 genes, <5e+04 counts and <15% mitochondrial reads were retained. Expression values were library size corrected to 10,000 reads per cell and log1p transformed, with Principal component analysis (PCA) performed on the scaled data for the top 2000 variable genes. Batch correction between NSC lines was performed on principal components using Harmony[14]. Uniform Manifold Approximation Projection embeddings, Nearest Neighbors were calculated on the top 15 harmony-corrected PCA dimensions using Seurat's RunUMAP(), FindNeighbors() functions and clusters were assigned using the

FindClusters() function with resolution=0.325. For cell type identification, cluster marker genes were calculated using a Wilcoxon Rank Sum test, and gene ontology term enrichment was performed of marker genes using the TopGO v2.52.0[15] R package. To compare cells against a large, published reference we accessed organoid cell type marker lists from Uzquiano et al. [16] and calculated average gene module expression for each single cell, subtracted by the aggregated expression of a random control set of features selected from the same average expression bins as the query genes[17]. Mean signature scores were then aggregated across clusters and visualized as a heatmap.

Neuroglial cell types were then subset and a new UMAP was calculated, as above. We next performed, zero-preserving imputation of the data using ALRA[18], removing pseudogenes and calculating differential expression between irradiated and control cells within each cell type using a Wilcoxon Rank Sum test. Gene ontology enrichment among the top upregulated genes (logFC >1.5) was calculated using topGO v2.52.0[15] and Gene set enrichment analyses on differentially regulated genes was performed using the fGSEA v4.4 R package[6], and gene sets were accessed from MsigDB[7]. The top 75 upregulated genes in neural and glial niches (Supplementary Fig. 5B) were taken as radiation signatures and enrichment was calculated using the AddModuleScore() Seurat function. For receptor-ligand analysis, cell type meta data and count data for the top 12500 most highly variable genes from remaining cells was input into CellphoneDB v5.0[11,19], and run using the deg_anlysis mode using differential expressed genes in ExNeurons as the input list. For circos plot visualization the Circlize v0.4.15 R package was used, and only those significant receptor-ligand interactions that involved ligands expressed by ExNeurons were included in the plot.

DNA and RNA extraction from COs

COs were dissociated. The RNA/DNA/Protein Purification Plus Kit (Norgen, #47700) was utilised to extract DNA and RNA, as per the manufacturer's instructions. For the extraction of DNA and RNA when working with organoids embedded in gelatine, extra wash steps were undertaken before processing.

Histological and immunofluorescence staining

Hematoxylin and eosin (H&E) staining was performed using standard protocols and slides were imaged using the EVOS XL core imaging system for brightfield images.

Immunofluorescence staining was performed using standard protocols. The following primary antibodies were used in the CO characterisation study: goat anti-Sox2 (1:500, Santa Cruz), rabbit anti-NeuN (1:200, Abcam), mouse anti-Nestin (1:200, Chemicon), rabbit anti- TBR1 (1:200, Abcam) and mouse anti-TUJ1 (1:200, Abcam). For irradiation experiments, the following antibodies were utilised: mouse anti-phospho-Histone-H2AX (1:250, Merck), mouse anti-8-Hydroxy-2'-deoxyguanosine (1:100, Abcam), rabbit anti-NF-kB p65 (1:100, Abcam), rabbit anti-DNMT1 (1:100, Abcam), rabbit anti-DNMT3A (1:200, Cell Signaling) and rabbit anti-DNMT3B (1:200,Abcam). The following secondary antibodies were used in the study: donkey anti-goat antibody Alexa Fluor 647 (1:200, Invitrogen™), donkey anti-rabbit Alexa Fluor 546 (1:200, Invitrogen™), donkey anti-mouse Alexa Fluor 488 (1:200, Invitrogen™), goat anti-rabbit Alexa Fluor 546 (1:200, Invitrogen™) , goat anti-mouse Alexa Fluor 488 (1:200, Invitrogen™), goat anti-mouse Alexa Fluor 546 (1:200, Invitrogen™), goat anti-rabbit Alexa Fluor 546 (1:200, Invitrogen™), goat anti-mouse Alexa Fluor 647 (1:200, Invitrogen™) and goat anti-rabbit 647 (1:200, Invitrogen™).

Confocal microscopy and quantification

Immunofluorescence images were acquired with the Zeiss 710 Laser Scanning confocal microscope. Images were captured with 20X and 63X magnification lenses depending on the nature of staining, and a series of Z-stack images were acquired for those that required assessment of signal dynamics. For images that required quantification, confocal calibration settings remained consistent across each staining set. Images were then quantified and processed in FIJI ImageJ. The region of interest was designated by creating a mask on the nuclei staining to extract the average intensity value. The mask threshold was kept automatic to distinguish between the background and the signal. The mean grey value was then calculated from the applied mask corresponding to the signal of interest. Five to ten images per sample, form three samples per condition, were captured for quantification. The relative mean fluorescence intensity (MFI) value was subsequently calculated using Microsoft Excel. To quantify colocalisation, the Manders' colocalisation coefficient (M1) value was calculated to determine the fraction of overlap between two signals. Manders' coefficient is a well-established measure which calculates the co-occurrence fraction of a fluorescent signal from one channel with that of another. M1 value ranges from 0 to 1, with 0 indicating total absence of colocalisation and 1 indicating perfect colocalisation[23]. Just Another Colocalization Plugin (JACoP) from Image J was used to carry out the M1 calculation from the region of interest. A

series of Z-stacks containing 8-12 images were processed at 63X magnification to calculate the colocalisation between signals in the set of images. The background was subtracted, and the noise was reduced with the despeckle tool to ensure the exclusion of artefacts from the calculation. Following the selection of nuclei of interest, z-stack images corresponding to these nuclei were determined, and the region of interest was isolated using the crop tool. The JACoP plugin created an automatic mask from the two selected channels of interest, and the M1 value was calculated, generating an overlap value from the signals analysed.

RT-qPCR

SuperScript III Reverse Transcriptase (Invitrogen™, #18080093) was used for the reverse transcription process as per the manufacturer's protocol. Random primers were mixed with RNA and diluted with PCR grade water, making up to 10 µL of solution. Primers were annealed at 65°C for at least 1 minute and then cooled at 4°C for 5 minutes. 5X FS Buffer, 0.1 M DTT and SuperScript III Reverse mix was added to the solution. The mixture was then incubated at 25°C for 5 minutes and incubated at 50°C for 30 minutes. Reaction time is increased to 55°C thereafter and finally, the inactivation step is carried out by heating at 70°C for 15 minutes. The cDNA was diluted to a concentration of 2.5 ng/µl using PCR grade water and stored in -20°C until further qPCR reactions were carried out. For the qPCR reaction, predesigned primers KiCqStart® SYBR® Green Primers (Sigma-Aldrich, # KSPQ12012) were used for gene expression analysis. To prepare the qPCR mix, add 5 µl of the forward and reverse primers with per 90 µl of PCR grade water for a final concentration of 10 µM. 96-well PCR plates (Applied Biosystems™, #4346906) were used to carry out the qPCR reaction with each well comprising of 1µl of the forward and reverse primer mix, 2 µl of diluted cDNA (5ng), 6 µl of PowerUp™ SYBR™ Green Master Mix (Applied Biosystems, # A25742) and 3 µl of PCR grade water. TAC1: forward primer (ATTCTGTGGCTTATGAAAGG), reverse primer (CATTGACACAAATGAAGCTG). PENK: forward primer (AACTGTCATTTCAGGTTCTG), reverse primer (TTTATGCACTTGGGGTAATAG). StepOnePlus™ Real-Time PCR System and StepOne Software (v2.3) were used to run the qPCR reaction. In addition, fast Cycling Mode (Primer Tm ≥60°C) was selected from the PowerUp™ SYBR® Green Master Mix manual for the qPCR thermal-cycling conditions. Cycle threshold (Ct) values were obtained from each gene run within a single reaction. ACTIN was designated as the housekeeping gene, and the cycle threshold (Ct) values for the gene were recorded with up to 40 cycles run. The Ct from genes of interest were normalised to that of the

housekeeping gene. Subsequent calculations were performed in Microsoft Excel to attain the ΔCt values from Ct(gene of interest)−Ct(housekeeping gene). ΔΔCt values were derived by calculating the ΔCt difference between the irradiated samples and the control. The Relative quantity (RQ) of gene expression was calculated using the $2^{(-\Delta\Delta Ct)}$. GraphPad Prism 9 was used to visualise data. ACTIN was used as the housekeeping gene for all qPCR analyses.

Statistical analysis using GraphPad Prism v9

Normalised signature scores, colocalisation, RT-qPCR, and immunofluorescence values were imported into GraphPad Prism version 9 for statistical analysis and visualisation. Unpaired t-tests were used to compare between two datasets, and analysis of variance (ANOVA) was carried out to investigate more than two groups. P-values for statistically significant results are represented in the graphs are as follows: $*P \leq 0.05$, $**P \leq 0.01$, $***P \leq 0.001$, $****P \leq 0.0001$. Error bars presented standard error of mean (SEM) or standard deviation (SD) depending on the nature of the analysis.

Data availability

All raw data is available on NIH GEO DataSets: DNA methylation: GSE264703; bulk RNAseq: GSE265797; spatial transcriptomics: GSE272334; scRNAseq data: GSE289218. All data contributing to figures is available as accompanying supplementary data.

References

1. Pidsley R, Y Wong CC, Volta M, Lunnon K, Mill J, Schalkwyk LC. A data-driven approach to preprocessing Illumina 450K methylation array data. *BMC Genomics*. May 01 2013;14:293. doi:10.1186/1471-2164-14-293
2. Jaffe A. *FlowSorted.Blood.450k: Illumina HumanMethylation data on sorted blood cell populations*. R package version 1.44.02024.
3. Gervin K, Hansen K. *FlowSorted.CordBloodNorway.450k: Illumina HumanMethylation data on sorted cord blood cell populations*. R package version 1.32.02024.
4. Hannon E, Dempster EL, Davies JP, et al. Quantifying the proportion of different cell types in the human cortex using DNA methylation profiles. *BMC Biol*. Jan 25 2024;22(1):17. doi:10.1186/s12915-024-01827-y
5. Zhang Y, Maksimovic J, Naselli G, et al. Genome-wide DNA methylation analysis identifies hypomethylated genes regulated by FOXP3 in human regulatory T cells. *Blood*. Oct 17 2013;122(16):2823-36. doi:10.1182/blood-2013-02-481788
6. Cvitic S, Novakovic B, Gordon L, et al. Human fetoplacental arterial and venous endothelial cells are differentially programmed by gestational diabetes mellitus, resulting in cell-specific barrier function changes. *Diabetologia*. Nov 2018;61(11):2398-2411. doi:10.1007/s00125-018-4699-7
7. de Witte LD, Wang Z, Snijders GLJL, et al. Contribution of Age, Brain Region, Mood Disorder Pathology, and Interindividual Factors on the Methylome of Human Microglia. *Biol Psychiatry*. Mar 15 2022;91(6):572-581. doi:10.1016/j.biopsych.2021.10.020
8. Grabovska Y, Mackay A, O'Hare P, et al. Pediatric pan-central nervous system tumor analysis of immune-cell infiltration identifies correlates of antitumor immunity. *Nat Commun*. Aug 28 2020;11(1):4324. doi:10.1038/s41467-020-18070-y
9. Newman AM, Steen CB, Liu CL, et al. Determining cell type abundance and expression from bulk tissues with digital cytometry. *Nat Biotechnol*. Jul 2019;37(7):773-782. doi:10.1038/s41587-019-0114-2
10. Xu S, Hu E, Cai Y, et al. Using clusterProfiler to characterize multiomics data. *Nat Protoc*. Nov 2024;19(11):3292-3320. doi:10.1038/s41596-024-01020-z
11. Kaufmann M, Schaupp AL, Sun R, et al. Identification of early neurodegenerative pathways in progressive multiple sclerosis. *Nat Neurosci*. Jul 2022;25(7):944-955. doi:10.1038/s41593-022-01097-3
12. Al-Dalahmah O, Argenziano MG, Kannan A, et al. Re-convolving the compositional landscape of primary and recurrent glioblastoma reveals prognostic and targetable tissue states. *Nat Commun*. May 04 2023;14(1):2586. doi:10.1038/s41467-023-38186-1
13. Chen WT, Lu A, Craessaerts K, et al. Spatial Transcriptomics and In Situ Sequencing to Study Alzheimer's Disease. *Cell*. Aug 20 2020;182(4):976-991.e19. doi:10.1016/j.cell.2020.06.038
14. Maynard KR, Collado-Torres L, Weber LM, et al. Transcriptome-scale spatial gene expression in the human dorsolateral prefrontal cortex. *Nat Neurosci*. Mar 2021;24(3):425-436. doi:10.1038/s41593-020-00787-0
15. Hao Y, Stuart T, Kowalski MH, et al. Dictionary learning for integrative, multimodal and scalable single-cell analysis. *Nat Biotechnol*. Feb 2024;42(2):293-304. doi:10.1038/s41587-023-01767-y
16. Korotkevich G, Sukhov V, Budin N, Shpak B, Artyomov MN, Sergushichev A. Fast gene set enrichment analysis. *bioRxiv*. 2016;doi:10.1101/060012
17. Liberzon A, Birger C, Thorvaldsdottir H, Ghandi M, Mesirov JP, Tamayo P. The Molecular Signatures Database (MSigDB) hallmark gene set collection. *Cell Syst*. Dec 23 2015;1(6):417-425. doi:10.1016/j.cels.2015.12.004
18. Miller BF, Huang F, Atta L, Sahoo A, Fan J. Reference-free cell type deconvolution of multi-cellular pixel-resolution spatially resolved transcriptomics data. *Nat Commun*. Apr 29 2022;13(1):2339. doi:10.1038/s41467-022-30033-z
19. Song L, Pan S, Zhang Z, Jia L, Chen WH, Zhao XM. STAB: a spatio-temporal cell atlas of the human brain. *Nucleic Acids Res*. Jan 08 2021;49(D1):D1029-D1037. doi:10.1093/nar/gkaa762

20. Efremova M, Vento-Tormo M, Teichmann SA, Vento-Tormo R. CellPhoneDB: inferring cell-cell communication from combined expression of multi-subunit ligand-receptor complexes. *Nat Protoc*. Apr 2020;15(4):1484-1506. doi:10.1038/s41596-020-0292-x
21. Kanemaru K, Cranley J, Muraro D, et al. Spatially resolved multiomics of human cardiac niches. *Nature*. Jul 2023;619(7971):801-810. doi:10.1038/s41586-023-06311-1
22. Vinel C, Rosser G, Guglielmi L, et al. Comparative epigenetic analysis of tumour initiating cells and syngeneic EPSC-derived neural stem cells in glioblastoma. *Nat Commun*. Oct 21 2021;12(1):6130. doi:10.1038/s41467-021-26297-6
23. Dunn KW, Kamocka MM, McDonald JH. A practical guide to evaluating colocalization in biological microscopy. *Am J Physiol Cell Physiol*. Apr 2011;300(4):C723-42. doi:10.1152/ajpcell.00462.2010
